# Integrating linkage mapping and GWAS reveals novel genetic architecture of seed weight in soybean (*Glycine max* L.)

**DOI:** 10.3389/fpls.2025.1711905

**Published:** 2026-01-05

**Authors:** Chunlei Zhang, Huilong Hong, Rongqiang Yuan, Kezhen Zhao, Bire Zha, Sobhi F. Lamlom, Xiaoyu Xi, Honglei Ren, Lijuan Qiu, Jiajun Wang

**Affiliations:** 1 Soybean Research Institute of Heilongjiang Academy of Agriculture Sciences, Harbin, China; 2 National Key Facility for Crop Gene Resources and Genetic Improvement, Institute of Crop Sciences, Chinese Academy of Agricultural Sciences, Beijing, China; 3 Plant Production Department, Faculty of Agriculture Saba Basha, Alexandria University, Alexandria, Egypt

**Keywords:** soybean, seed weight, QTL mapping, GWAS, SLAF-seq, candidate genes, molecular breeding

## Abstract

**Objective:**

Seed weight is a key factor in soybean yield and value, but its genetic basis and environmental stability are not fully understood. Despite many QTL studies, there’s a lack of integration between bi-parental linkage mapping and diverse germplasm association analysis. We hypothesized that combining high-resolution QTL mapping in recombinant inbred lines with GWAS in natural populations could identify both population-specific and broadly segregating seed weight loci, aiding in candidate gene discovery for breeding.

**Methods:**

We integrated biparental QTL mapping with genome-wide association studies (GWAS) to comprehensively dissect the genetics of hundred-seed weight (HSW). A recombinant inbred line population of 325 F_2_:_5_ lines from Qihuang 34 × Dongsheng 16 was phenotyped across three environments and genotyped using SLAF-seq, generating a high-density genetic map with 6,297 SNP markers spanning 2,945.26 cM (0.47 cM resolution). Simultaneously, 348 diverse soybean accessions underwent whole-genome resequencing (10× coverage), yielding 1,882,531 SNPs for association analysis across two years.

**Results:**

QTL mapping identified 11 significant loci explaining 2.47-8.59% of phenotypic variance, with broad-sense heritability of 0.78. The major-effect QTL *qHSW-19-4* (44.84-44.85 Mb, LOD = 9.72) demonstrated unprecedented 11.4 kb mapping precision. GWAS independently detected six genome-wide significant associations (P < 1 × 10^–8^), including a stable chromosome 19 peak at 45.28 Mb (P = 2.06 × 10^–^²³) explaining 15.3-18.7% of variance. Remarkably, this GWAS signal co-localized within 580 kb of *qHSW-19-4*, providing robust cross-population validation of chromosome 19 as a major seed weight regulatory region. Functional analysis of 44 candidate genes, validated by quantitative RT-PCR across seed developmental stages, identified four high-priority candidates: *Glyma.19G195400* (cell wall invertase, 2.7-fold upregulation in large-seeded parent, r = 0.68 with HSW), *Glyma.19G194300* (PEBP/Dt1 family protein), *Glyma.19G193400* (bZIP transcription factor), and *Glyma.06G095100* (Myb DNA-binding domain).

**Novelty and conclusions:**

This first integrated QTL-GWAS analysis for soybean seed weight reveals both major-effect loci and polygenic architecture, providing validated molecular markers and candidate genes for breeding programs targeting yield improvement.

## 1 Introduction

Soybean (*Glycine max* L. Merr.) represents one of the most economically important legume crops globally, serving as a fundamental source of plant-based protein and vegetable oil for both human consumption and industrial applications (Tian et al., 2025). The crop’s significance has steadily increased over recent decades, with global production reaching approximately 350 million metric tons annually, driven by expanding demand from food processing, livestock feed, and biofuel industries (Faostat, 2016; Popescu, 2024). Among the various agronomic traits that determine soybean productivity, hundred-seed weight stands out as a critical yield component that directly influences both grain yield potential and market value (Schünemann et al., 2024).

Hundred-seed weight (HSW) exhibits substantial genetic variation among soybean genotypes and serves as a primary determinant of final grain yield. Previous research has established positive correlations between individual seed weight and overall productivity, with heavier seeds generally contributing to higher per-plant yields when other yield components remain constant (Wang et al., 2025). Beyond yield implications, seed weight significantly affects seed quality attributes, including germination rate, seedling vigor, and stress tolerance during establishment (Yuan et al., 2024). Furthermore, seed size requirements vary considerably among different end-use applications, with large-seeded varieties preferred for direct human consumption products such as edamame and tofu, while smaller seeds are typically utilized for oil extraction and animal feed formulations (Place et al., 2011; Wang et al., 2025).

The genetic architecture underlying seed weight control in soybean has been extensively investigated through quantitative trait loci (QTL) mapping studies, revealing complex polygenic inheritance patterns. Comprehensive surveys of published literature indicate that seed weight QTLs have been detected across all twenty soybean chromosomes, with effect sizes ranging from minor contributions explaining less than 5% of phenotypic variance to major loci accounting for more than 20% of trait variation (Duc et al., 2023; Kumar et al., 2023; Zhang et al., 2021). Notable examples of stable, major-effect QTLs include the chromosome 17 locus *qSw17-1*, which consistently demonstrates significant effects across multiple genetic backgrounds and environmental conditions (Kato et al., 2014). Meta-analyses of seed weight QTL studies have identified several hotspot regions on chromosomes 1, 4, 6, 13, 15, and 20, where multiple independent studies have reported significant associations (Zhang et al., 2021). However, the molecular mechanisms governing seed size determination remain incompletely understood compared to model systems such as rice and *Arabidopsis* (Duan et al., 2015; Han et al., 2008; Su et al., 2011).

Despite the abundance of reported seed weight QTLs in soybean, several critical knowledge gaps persist. First, chromosome 19 has received limited attention in seed weight genetics. SoyBase QTL database searches reveal only sporadic reports of seed weight associations in this chromosomal region and no consistently validated major-effect loci. Recent genome-wide association studies (GWAS) using large germplasm collections have similarly not identified strong signals in the central region of chromosome 19 (Duc et al., 2023; Kumar et al., 2023; Zhang et al., 2021), suggesting that valuable genetic variation in this region may remain underexploited. Second, the functional validation of candidate genes underlying seed weight QTLs remains limited, with only a handful of genes functionally characterized in soybean, including the *ln* (narrow leaf) gene influencing both seed number per pod and individual seed size (Schmutz et al., 2010), and the *PP2C-1* gene encoding a protein phosphatase that regulates seed size through metabolic pathway modulation (Jeong et al., 2012). Third, traditional QTL mapping approaches using low to moderate marker densities (typically 100–500 markers) often achieve limited genetic resolution, constraining precise localization and subsequent gene identification efforts (Lee et al., 1996). High-density molecular marker technologies such as specific-locus amplified fragment sequencing (SLAF-seq) offer significant advantages by providing thousands of evenly distributed markers across the genome, enabling more precise QTL localization and improved detection power for moderate-effect loci (Collard et al., 2005).

To address these critical gaps, the present study pursued an integrated genetic mapping strategy combining high-density SLAF-seq-based QTL mapping in a large RIL population with whole-genome resequencing-based GWAS in a diverse natural germplasm panel. Specifically, our objectives were to: (1) construct a high-density genetic linkage map for the Qihuang 34 × Dongsheng 16 RIL population to enable high-resolution QTL detection; (2) identify and characterize seed weight QTLs through multi-environment phenotypic evaluation across contrasting northern (Harbin) and southern (Sanya) Chinese production environments to assess environmental stability and G×E interactions; (3) conduct GWAS using 348 diverse soybean accessions genotyped with whole-genome resequencing to identify broadly segregating seed weight variants and validate RIL-derived QTLs; (4) integrate linkage and association mapping results to identify co-localized genomic regions representing high-confidence seed weight regulatory loci; (5) perform comprehensive functional annotation of candidate genes within validated regions and prioritize candidates based on biological relevance to seed development; and (6) validate expression patterns of priority candidate genes across seed developmental stages in parental lines to establish genotype-phenotype correlations and identify the most promising targets for functional characterization. By integrating complementary genetic mapping approaches, multi-environment phenotyping, and gene expression validation, this study provides the most comprehensive dissection of soybean seed weight genetics to date, delivering validated molecular markers, characterized candidate genes with expression evidence, and genomic resources to accelerate breeding for improved seed weight and yield across diverse global production systems.

## 2 Materials and methods

### 2.1 Plant materials

#### 2.1.1 RIL population development

A recombinant inbred line (RIL) population consisting of 325 F_2_:_5_ lines was developed from a cross between two soybean (*Glycine max* L. Merr.) cultivars. The female parent, Qihuang 34 (You Chu NO.4/86573-16), is a high-yielding cultivar selected by the Crop Research Institute of Shandong Academy of Agricultural Sciences. The male parent, Dongsheng 16 (Jia Nong NO.1/(Hai6055/Dong Sheng NO.1)), was developed by the Agricultural Technology Center of Northeast Institute of Geography and Agroecology, Chinese Academy of Sciences. Qihuang 34 exhibits a characteristically large seed size (hundred-seed weight: 22.3 ± 0.8 g, averaged across three environments), an indeterminate growth habit, yellow seed coat color, and maturity group III classification. Agronomic performance trials conducted across multiple locations in Shandong Province demonstrated average yield potential of 3,500 kg/ha with excellent seed quality characteristics including 42.5% protein content and 19.8% oil content on a dry weight basis.

The male parent, Dongsheng 16, moderate seed size (hundred-seed weight: 19.8 ± 0.6 g), Dongsheng 16 exhibits superior cold tolerance during germination and early vegetative growth, rapid canopy closure, determinate growth habit, black hilum color, and maturity group I classification suitable for high-latitude production regions (48-50°N). Multi-location trials in Heilongjiang Province demonstrated average yield of 2,800 kg/ha with excellent adaptation to short growing seasons (105–110 days from planting to physiological maturity) and resistance to prevalent diseases including soybean mosaic virus and bacterial pustule.

#### 2.1.2 Natural population for GWAS

A diverse germplasm panel comprising 348 soybean accessions was assembled for genome-wide association analysis, representing the genetic diversity of northern spring soybean production zones in China. The collection included: 87 accessions from Heilongjiang Province, 128 from Jilin Province, 29 from Liaoning Province, 11 from Inner Mongolia, 4 from Xinjiang, and 89 from other northern regions. This panel encompassed elite cultivars, improved varieties, landraces, and wild soybean (*Glycine soja*) accessions to maximize allelic diversity.

### 2.2 Field experiments and phenotypic evaluation

#### 2.2.1 Experimental design

Both the RIL population and natural diversity panel were evaluated across two locations and multiple growing seasons: (1) Harbin, Heilongjiang Province (45.86°N, 126.79°E) from May to October 2023, and (2) Yazhou, Hainan Province (18.36°N, 109.17°E) from October 2023 to May 2024. An additional evaluation of the RIL population was conducted in Harbin in 2022 for 100-seed weight. Field trials were established using a randomized complete block design (RCBD) with three biological replicates. Each plot consisted of a single 3-m row with 5-cm plant spacing, 65-cm ridge width in Harbin, and 50-cm ridge width in Hainan. Standard agronomic management practices appropriate for each location were implemented, including fertilization, irrigation, and pest control.

#### 2.2.2 Phenotypic data collection

Hundred-seed weight (HSW) served as the primary phenotypic trait measured in this study. At physiological maturity (R8 growth stage, identified when approximately 95% of pods reached mature pod color with characteristic drying and browning), individual plots were hand-harvested by cutting plants at ground level and bundling by plot. To minimize sampling bias and ensure representative seed sampling, we excluded the outermost five plants on each end of each row (border plants that may be affected by edge effects or inter-plot competition), leaving approximately 50 plants in the middle section of each 3-meter row for sampling. From these central plants, 15 representative plants were randomly selected across the length of the harvested plot section, representing approximately 30% of the harvestable plot area and capturing spatial variation within plots. All pods from these 15 selected plants were hand-threshed, and seeds were cleaned to remove debris and plant material. Seeds were air-dried at ambient temperature (20-25°C) with regular turning for 7–10 days until reaching equilibrium moisture content, verified by electronic moisture meter to be 12-14% moisture content on a wet basis. From the bulk seed lot per plot (typically 800-1,200 seeds from 15 plants), three independent 100-seed samples were randomly counted using a model SLY-B electronic seed counter (Zhejiang Top Instrument, China) to ensure accuracy and minimize counting errors. Each 100-seed sample was weighed using a Mettler Toledo XS205 analytical balance (precision 0.01 mg, readability 0.1 mg; Mettler-Toledo International Inc., Switzerland), which was calibrated daily using certified reference weights. The three 100-seed weight measurements per plot were averaged to produce the plot-level mean hundred-seed weight value, with the coefficient of variation among the three subsamples calculated to assess within-plot sampling variability. Plots showing CV >2.5% among subsamples were resampled and remeasured to ensure data quality. The plot-level mean hundred-seed weight values were used as the phenotypic data for QTL mapping, representing the experimental unit for statistical analysis. This sampling protocol provided robust estimates of plot-level seed weight while accounting for within-plot variation in seed size, which may arise from positional effects, variable pod positions on plants, or microenvironmental heterogeneity.

#### 2.2.3 Phenotypic data analysis

Phenotypic data quality was assessed through descriptive statistics, including mean, range, standard deviation (SD), variance, skewness, kurtosis, and coefficient of variation (CV). Normality of trait distributions was tested using the Shapiro-Wilk test. Best linear unbiased predictions (BLUPs) were calculated across environments using the lme4 package (Bates et al., 2015) in R v4.2.0 with the following mixed linear model:

y_ijk=μ+G_i+E_j+R_k(E_j)+GE_ij+ϵ_ijk where y_ijk is the phenotypic observation, μ is the overall mean, G_i is the genotype effect (random), E_j is the environment effect (fixed), R_k(E_j) is the replication effect nested within environment (random), GE_ij is the genotype-by-environment interaction effect (random), and ϵ_ijk is the residual error.

Broad-sense heritability (H^2^) was estimated using variance components:

${\text{H}}^{2}={\text{σ}}^{2}\_\text{G}/({\text{σ}}^{2}\_\text{G}+{\text{σ}}^{2}\_\text{GE}/\text{e}+{\text{σ}}^{2}\_\text{ϵ}/\text{re})$ where σ²_G is genotypic variance, σ^2^_GE is genotype-by-environment interaction variance, σ^2^_ϵ is residual variance, e is the number of environments, and r is the number of replications.

Pearson correlation coefficients were calculated to examine phenotypic relationships among traits using the corrplot package v0.92 (Wei and Simko, 2021) in R. Statistical significance was determined at P < 0.05 (*), P < 0.01 (), P < 0.001 (*), and P < 0.0001 (****).

### 2.3 DNA extraction and genotyping

Fresh leaf tissue was collected at the third trifoliate stage (V3 developmental stage) from each accession and immediately frozen in liquid nitrogen. Genomic DNA was extracted using the FastPure^®^ Plant DNA Isolation Mini Kit (DC104, Vazyme Biotech Co., Nanjing, China) following the manufacturer’s protocol. DNA quality was evaluated by 1.0% agarose gel electrophoresis, and concentration was determined using a NanoDrop OneC spectrophotometer (Thermo Fisher Scientific, Wilmington, DE, USA). Samples with A260/A280 ratios between 1.8 and 2.0 and concentrations ≥50 ng/μL were selected for library construction.

### 2.4 SLAF-seq genotyping and SNP calling

Specific-locus amplified fragment sequencing (SLAF-seq) was performed for high-density genotyping of the RIL population. Genomic DNA samples (n=327: 325 RILs + 2 parents) were normalized to 50 ng/μL concentration based on Qubit fluorometric quantification. For each sample, 500 ng of genomic DNA (10 μL at 50 ng/μL) was digested with RsaI and HaeIII as described above. Following complete digestion confirmed by analytical gel electrophoresis of 5% of each digestion reaction, digested fragments underwent 3′-end A-tailing by addition of dATP and Klenow Fragment (3′→5′ exo^–^) DNA polymerase, creating 3′-A overhangs on all fragments compatible with T-overhang adapter ligation (Schmutz et al., 2010), to optimize fragment distribution and density. Following enzymatic digestion, fragments were subjected to 3′-end A-tailing and ligated with dual-index adapters. Target fragments (300–500 bp) were size-selected through gel electrophoresis and PCR amplified to enrich adapter-ligated fragments. Libraries were sequenced on the Illumina HiSeq 2500 platform using paired-end 150 bp sequencing chemistry. High-quality filtered reads were aligned to the soybean reference genome *Glycine max* Wm82.a2.v1 using the BWA-MEM algorithm with default parameters optimized for short-read alignment, achieving an average alignment rate across all samples. SLAF tags were defined as clusters of aligned sequences with ≥95% sequence identity (allowing up to 5% sequence divergence from the reference to accommodate sequencing errors and true SNPs) and mapped to consistent genomic locations within 100 bp windows. Only SLAF tags meeting stringent coverage criteria were retained for subsequent analysis: average sequencing depth ≥5× across all samples (ensuring adequate confidence for genotype calling), detection in >80% of samples (requiring <20% missing data), and containing ≥5 high-quality single-nucleotide polymorphisms (SNPs) between parents (providing sufficient information content for genetic mapping). SNP calling employed SAMtools mpileup and BCFtools call with a multiallelic calling model, identifying putative SNP positions where both parents showed homozygous genotypes differing from each other and read depth ≥5× in both parents. SNPs were further filtered to retain only biallelic SNPs (excluding multi-allelic sites complicated by alignment artifacts or genome duplications) with minor allele frequency (MAF) ≥0.05 in the RIL population (ensuring adequate segregation for linkage analysis) and missing data <10% across RILs. This stringent filtering pipeline yielded high-confidence SNP datasets for genetic map construction and QTL analysis.

### 2.5 Whole-genome resequencing for natural population

The 348 accessions in the natural population underwent whole-genome resequencing. Libraries were constructed using the standard Illumina paired-end protocol with 350-bp insert size. Genomic DNA (1 μg) was fragmented to 250–350 bp using a Bioruptor ultrasonicator (Diagenode, Denville, NJ, USA). Fragmented DNA was end-repaired, A-tailed, ligated with dual-indexed adapters, and amplified by PCR. Libraries were sequenced on an Illumina NovaSeq 6000 platform at Novogene Bioinformatics Technology Co., Ltd. (Beijing, China), targeting an average sequencing depth of 10× per accession. Raw sequencing data were quality-controlled using FastQC v0.11.9 (Andrews et al., 2010) and processed with Trimmomatic v0.39 to remove adapters and low-quality bases (Q < 20). Clean reads were mapped to the Wm82.a2.v1 reference genome using BWA-MEM v0.7.17 with default parameters. SAMtools v1.12 was used to convert SAM files to sorted BAM format, and PCR duplicates were removed using Picard MarkDuplicates v2.25.0 (http://broadinstitute.github.io/picard/). SNP calling was performed using GATK v4.2.0 following the Best Practices workflow. Variant filtration applied the following criteria: mapping quality (MQ) ≥30, quality by depth (QD) ≥2.0, Fisher strand bias (FS) ≤60.0, strand odds ratio (SOR) ≤3.0, mapping quality rank sum test (MQRankSum) ≥-12.5, and read position rank sum test (ReadPosRankSum) ≥-8.0. Final SNP filtering retained biallelic SNPs with call rate ≥95%, MAF ≥0.05, and Hardy-Weinberg equilibrium P-value >1 × 10^–6^. SNP annotation was conducted using SnpEff v5.0based on Wm82.a2.v1 gene models. A total of 1,882,531 high-quality SNPs distributed across 20 chromosomes were obtained for subsequent analysis.

### 2.6 Genetic map construction and QTL analysis

#### 2.6.1 High-density genetic linkage map

Genetic linkage map construction for the RIL population was performed using HighMap software (Liu et al., 2014) employing a modified Kosambi mapping function. The linkage grouping was conducted with a logarithm of odds (LOD) threshold of≥3.0 and a recombination fraction of <0.4. Markers were ordered within linkage groups using the enhanced Monte Carlo multipoint maximum likelihood algorithm implemented in HighMap. Map quality was assessed by evaluating marker collinearity between the genetic map and the physical genome. Spearman rank correlation coefficients were calculated for each linkage group to quantify the concordance between genetic and physical marker orders, with values greater than 0.95 indicating high-quality map construction. The final high-density genetic map consisted of 6,297 SNP markers spanning 2,945.26 cM across 20 linkage groups corresponding to the 20 soybean chromosomes. The average genetic distance between adjacent markers was 0.47 cM, significantly denser than conventional genetic maps, providing enhanced resolution for QTL detection.

#### 2.6.2.QTL mapping

QTL analysis was conducted using IciMapping v4.2 software (Meng et al., 2015) with the Inclusive Composite Interval Mapping of Additive QTL (ICIM-ADD) method. This approach combines the strengths of interval mapping and composite interval mapping while controlling background genetic variation through stepwise regression. The scanning parameters were set as follows: 1-cM walking speed, probability in stepwise regression (PIN) of 0.001, and a minimum LOD threshold determined by 1,000 permutation tests at α = 0.05 (typically LOD ≥2.5-3.0).

### 2.7 Population genetic structure analysis

The population structure of the natural diversity panel was assessed using principal component analysis (PCA) implemented in PLINK v1.9. To minimize the influence of linkage disequilibrium (LD), SNPs were pruned using the “–indep-pairwise 50 10 0.2” command, which retains one SNP per 50-SNP window if pairwise r^2^ values exceed 0.2. PCA was performed on the pruned SNP dataset, and the top 10 principal components (PCs) were extracted. Scree plots of eigenvalues were examined to determine the number of informative PCs. The first two PCs, explaining 50.4% and 49.6% of the genetic variance, respectively, revealed a continuous population structure with gradual differentiation corresponding to chromosome number, indicating the absence of discrete subpopulations. A kinship matrix was calculated using the VanRaden method, as implemented in TASSEL v5.2.80, to account for cryptic relatedness among accessions. Genome-wide LD decay was estimated by calculating squared correlation coefficients (r^2^) between all pairwise SNPs within 5 Mb using PopLDdecay v3.41 (. The LD decay distance was defined as the physical distance at which r^2^ declined to 0.1 or to half its maximum value. The average LD decay distance was estimated at 1.58 Mb, with r^2^ reaching 0.1 at approximately 4.36 Mb, providing guidance on the candidate gene window size in GWAS. Chromosome-specific LD heatmaps were generated using the LDheatmap package v1.0–4 in R to visualize local LD patterns.

### 2.8 Genome-wide association study

Genome-wide association analysis for 100-seed weight was performed using a compressed mixed linear model (CMLM) implemented through the FarmCPU (Fixed and random model Circulating Probability Unification) algorithm in the rMVP package v1.0.5, with general linear model (GLM) and mixed linear model (MLM) applied for comparative validation using TASSEL v5.2.80. The statistical models were formulated as: GLM: y = Xβ + Zu + e, where y represents the phenotype vector (BLUP values across environments), X is the design matrix for fixed effects (SNP genotypes and top 3 principal components), β is the fixed effect vector, Z is the incidence matrix, u is the SNP effect, and e is the residual error; and MLM: y = Xβ + Zu + Kμ + e, where K is the kinship matrix calculated using the VanRaden method and μ represents random polygenic effects following N(0, Kσ^2^_g). The first three principal components (PC1-PC3), explaining 75.3% of genetic variation, were incorporated as fixed-effect covariates to control for population stratification, and the kinship matrix was included to account for cryptic relatedness. The genome-wide significance threshold was determined using Bonferroni correction adjusted for effective independent tests estimated by the simpleM method, yielding P < 1 × 10^–8^ (corresponding to -log_10_(P) > 8.0), while a suggestive threshold of P < 1 × 10^–5^ (-log_10_(P) > 5.0) was applied to identify additional candidate loci. Model appropriateness was evaluated using quantile-quantile (Q-Q) plots, with genomic inflation factors (λ) calculated as λ = median(χ^2^_observed)/0.456, where values between 0.95 and 1.05 indicated adequate control of population structure. Manhattan plots displaying -log_10_(P) values across the 20 chromosomes were generated using CMplot v4.0.0, and phenotypic variance explained (PVE) by each significant SNP was calculated as PVE = (SSR_full - SSR_reduced)/SST × 100%, where SSR_full and SSR_reduced represent sum of squared residuals for models with and without the focal SNP, respectively. Candidate genes within ±200 kb flanking windows of lead SNPs were extracted from the *Glycine max* Wm82.a2.v1 genome annotation (Phytozome v13, SoyBase), and functional annotation was performed using Gene Ontology (GO) terms, KEGG pathways, and InterPro domains, with enrichment analysis conducted using agriGO v2.0 FDR-adjusted P < 0.05). Nonsynonymous SNPs within candidate genes were annotated using SnpEff v5.0 (Shi et al., 2024). Their functional impact was predicted using SIFT (Sorting Intolerant From Tolerant) analysis (Kumar et al., 2009), with scores <0.05 classified as deleterious, while allelic effects were estimated as the difference in least-squares means between homozygous genotype classes adjusted for population structure.

### 2.9 Integration of QTL and GWAS results

QTL confidence intervals identified through linkage mapping were compared with GWAS peaks to identify overlapping genomic regions. Co-localization was defined when: (1) GWAS significant SNPs fell within QTL confidence intervals, or (2) physical distances between QTL peak positions and GWAS lead SNPs were ≤1.5 Mb (based on average LD decay distance). Co-localized regions were prioritized as high-confidence loci for candidate gene mining. Co-localized loci were visualized using integrated Manhattan plots overlaid with QTL confidence intervals, and regional association plots were generated for key loci using LocusZoom (Pruim et al., 2010).

### 2.10 Candidate gene identification and annotation

Candidate genes within significant QTL/GWAS regions were extracted based on physical coordinates using a ±200 kb window (or QTL confidence interval boundaries) flanking peak SNPs, consistent with the estimated LD decay distance. Gene models were retrieved from the Wm82.a2.v1 annotation (*Glycine_max*_v2.1) available at Phytozome v13 (https://phytozome-next.jgi.doe.gov/) and SoyBase (www.soybase.org). GO and KEGG enrichment analyses were conducted using agriGO v2.0 (Khaje Ghiassi et al., 2022) with Fisher’s exact test and Benjamini-Hochberg false discovery rate (FDR) correction (P_adj < 0.05). Non-synonymous SNPs (nsSNPs) within coding sequences of candidate genes were identified for both the RIL parents (DS16 and QH34) and the natural population using SnpEff v5.0 annotation. The functional impact of amino acid substitutions was predicted using SIFT (Sorting Intolerant From Tolerant) scores (Kumar et al., 2009), with scores <0.05 indicating deleterious effects. Protein domain conservation and variant positions were examined using the Conserved Domain Database (CDD) at NCBI (Wang et al., 2023).

### 2.11 Quantitative real-time PCR analysis

For validating gene expression, we collected samples from the parental lines Qihuang 34 (QH34) and Dongsheng 16 (DS16) during seed development. We collected developing seeds at 15, 25, 35, and 45 days after flowering (DAF), representing early embryogenesis (15 DAF), the rapid cell division and expansion phases (25–35 DAF), and seed maturation (45 DAF), when final seed weight is primarily determined. For each genotype and time point combination, three biological replicates were collected, each consisting of developing seeds harvested from three independent plants within the same plot, spatially separated by at least 1 meter to minimize non-independence due to microenvironmental similarity. From each of the three plants per biological replicate, 10–15 developing seeds (total 30–45 seeds per biological replicate) were collected from pods located at mid-canopy positions (nodes 6–10 on the main stem) to standardize developmental timing and minimize positional effects on gene expression. Pods were selected based on visual assessment of developmental stage, with pod length and seed size serving as indicators. Seeds were immediately dissected from pods in the field, frozen in liquid nitrogen within 30 seconds of pod opening to rapidly quench enzymatic activity and preserve RNA integrity, and stored at -80°C until RNA extraction (within 2 weeks of collection). We extracted total RNA from 100 mg of ground seed tissue using the FastPure Plant Total RNA Isolation Kit (Vazyme, China). We assessed RNA quality using NanoDrop spectrophotometry (A260/A280 = 1.8-2.1) and agarose gel electrophoresis. We synthesized first-strand cDNA from 1 μg of RNA using HiScript III RT SuperMix (Vazyme) and diluted it 1:10 for analysis. We designed gene-specific primers for eight candidate genes using Primer Premier 5.0, targeting 100–200 bp amplicons with melting temperatures between 58-62 °C. We verified primer specificity using BLAST and melting curve analyses. We used β-tubulin (*Glyma.05G157200*) as the reference gene (**Supplementary Table S1**). We conducted qRT-PCR using ChamQ Universal SYBR qPCR Master Mix (Vazyme) on a CFX96 Touch system (Bio-Rad). Our reactions (20 μL) contained 10 μL SYBR Master Mix, 0.4 μL of each primer (10 μM), 2 μL cDNA, and 7.2 μL nuclease-free water. The thermal cycling conditions included 95°C for 30 seconds, followed by 40 cycles at 95°C for 10 seconds and 60°C for 30 seconds, and then melting curve analysis. We ran all samples in technical triplicates with no-template controls. We calculated relative expression levels using the 2^(-ΔΔCt) method, normalized to β-tubulin, with the high-seed-weight group at 15 DAF as the calibrator. We analyzed data by two-way ANOVA with Tukey’s HSD *post-hoc* tests. We used Pearson correlation analysis to examine relationships between gene expression and seed weight phenotypes, using R software (v4.3.0, p < 0.05).

### 2.12 Statistical analysis and data visualization

All statistical analyses were performed using R v4.2.0 (Giorgi et al., 2022). Phenotypic data were tested for normality using the Shapiro-Wilk test, and appropriate transformations (log, square root, or Box-Cox) were applied when necessary. Pearson correlation coefficients were calculated for trait relationships, with significance tested at P < 0.05. Analysis of variance (ANOVA) was conducted using the aov() function, followed by *post-hoc* comparisons using Tukey’s HSD test when appropriate. Significance levels are indicated as: ns, not significant (P > 0.05); * P < 0.05; ** P < 0.01; *** P < 0.001; **** P < 0.0001.

## 3 Results

### 3.1 Phenotypic variation and distribution analysis

**Figure 1:**
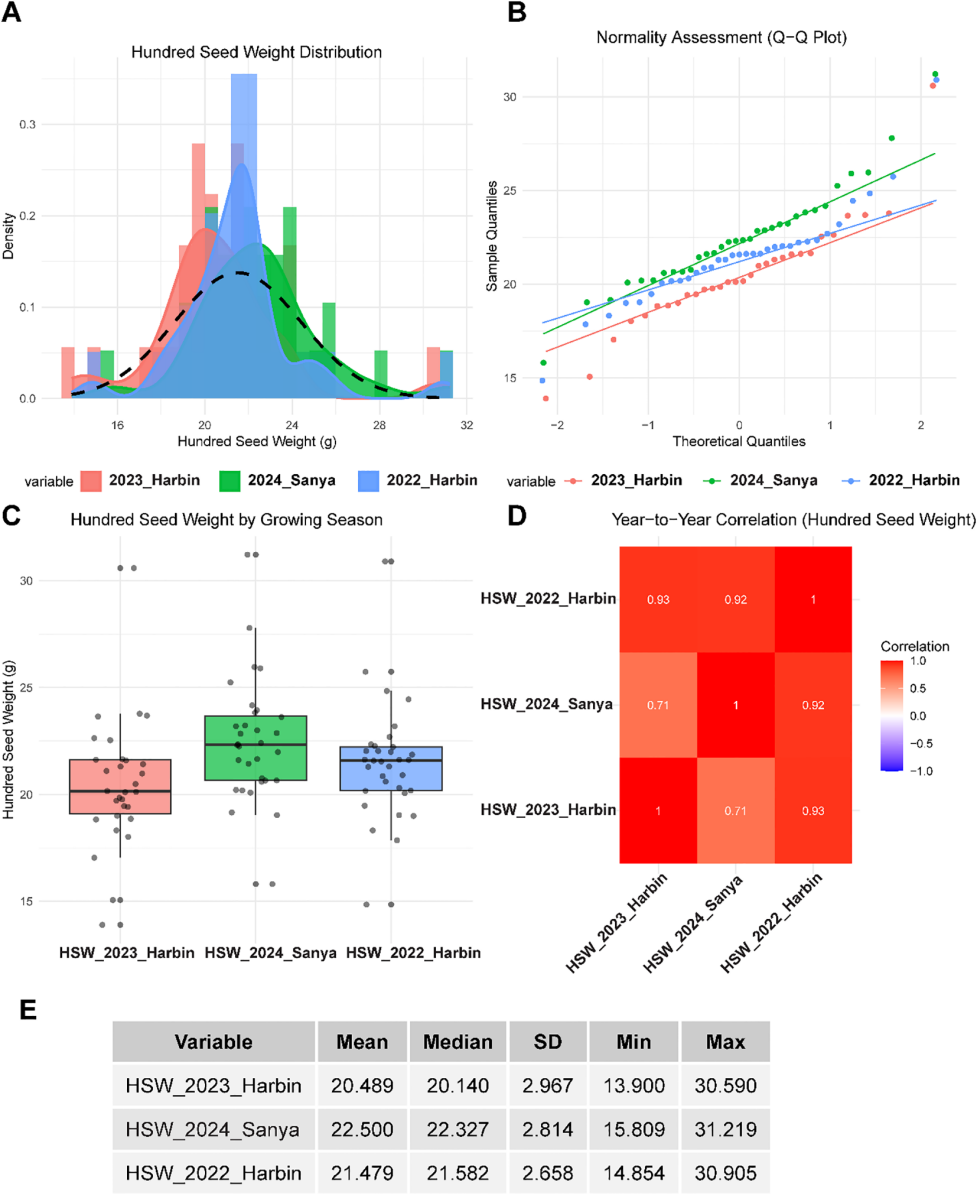
Phenotypic distribution and correlation analysis of hundred seed weight across multi-environment field trials. **(A)** Frequency distribution histograms with overlaid normal density curves (dashed black line) showing hundred seed weight variation across three environments: 2023 Harbin (red), 2024 Sanya (green), and 2022 Harbin (blue). **(B)** Quantile-quantile (Q-Q) plots assessing normality of hundred seed weight distributions for each environment, with theoretical normal quantiles plotted against sample quantiles. Points following the diagonal line indicate normal distribution. **(C)** Box plots displaying hundred seed weight distribution by environment, showing median (center line), interquartile range (box), whiskers (1.5× IQR), and individual data points (dots). Outliers are shown as points beyond the whiskers. **(D)** Correlation heatmap showing Pearson correlation coefficients between hundred seed weight measurements across the three environments. Color intensity indicates the strength of the correlation (red = positive, blue = negative), with numerical values displayed in each cell. **(E)** Descriptive statistics table summarizing key parameters for hundred seed weight in each environment.

The field experiments conducted across multiple locations and years revealed significant phenotypic variation in HSW, highlighting the quantitative nature of this key agronomic trait (**Figure 1**). HSW showed continuous variation, with means of 20.489 g in 2023 Harbin, 22.500 g in 2024 Sanya, and 21.479 g in 2022 Harbin. The standard deviations remained relatively consistent across environments (2.967 g in 2023 Harbin, 2.814 g in 2024 Sanya, and 2.658 g in 2022 Harbin), indicating stable genetic variation despite differing environmental conditions. Distribution analysis demonstrated that HSW approximately followed normal distributions across all three environments (**Figure 1A-B**), with histograms displaying characteristic bell-shaped curves overlaid by fitted normal density curves. The Q-Q plots (**Figure 1B**) confirmed the normality assumption for most data points, although some deviations were observed in the tails, particularly for extreme values. This near-normal distribution suggests that HSW is controlled by multiple genes with additive effects, making it suitable for quantitative genetic analysis. A comprehensive analysis across the three environments revealed significant effects of location and year on HSW performance (**Figure 1C-E**). The 2024 Sanya winter season provided the most favorable conditions for seed development, with the highest mean HSW (22.500 g) and median (22.327 g), compared to 2022 Harbin (mean: 21.479 g, median: 21.582 g) and 2023 Harbin (mean: 20.489 g, median: 20.140 g). Box plot analysis (**Figure 1C**) showed that the 2024 Sanya environment also had the widest interquartile range, indicating that the subtropical winter conditions permitted the maximum expression of genetic potential for seed size. Correlation analysis among environments revealed strong positive relationships (**Figure 1D**), with the highest correlation between the two Harbin seasons (2022 and 2023: r = 0.93), reflecting consistent genotype performance across years at the same site. Cross-location correlations were moderately high, with r = 0.71 between 2023 Harbin and 2024 Sanya and r = 0.92 between 2022 Harbin and 2024 Sanya. These strong positive correlations suggest good genotype stability across different environments and indicate that HSW is a reliable trait for selection under varying growing conditions, although the varying strength of correlations underscores the need for multi-environment testing for comprehensive genetic evaluation.

### 3.2 Phenotypic analysis of natural soybean population

Phenotypic evaluation of HSW across 348 soybean accessions revealed substantial genetic variation and environmental plasticity across two growing seasons (**Table 1**). In the 2023 Harbin environment, SW ranged from 6.35 to 59.53 g, with a population mean of 19.97 ± 4.62 g (mean ± SD). The trait exhibited a CV of 0.23 (23%), indicating moderate phenotypic diversity suitable for genetic dissection. The distribution showed strong positive skewness (2.36) and high kurtosis (18.98), suggesting a right-tailed distribution, with several accessions carrying alleles for large seed size (>40 g), while the majority clustered in the lower to intermediate range. This non-normal distribution pattern is characteristic of traits under directional selection during soybean domestication and modern breeding, where large-seeded types have been preferentially selected but remain relatively rare in northern spring soybean germplasm adapted to short growing seasons. In the 2024 Hainan environment, SW ranged from 9.51 to 40.22 g, with an increased population mean of 22.27 ± 4.71 g, representing a 11.5% increase relative to 2023. The CV remained similar at 0.21 (21%), indicating consistent relative variation despite the shift in mean. Notably, the 2024 distribution exhibited substantially reduced skewness (0.40) and kurtosis (1.54), approaching a near-normal distribution. This shift suggests that the Hainan environment (characterized by higher temperatures, more extended photoperiod, and different soil conditions) moderated extreme phenotypes, potentially through G×E interactions affecting seed filling duration or source-sink relationships. The reduction in maximum seed weight from 59.53 g to 40.22 g (-32.4%) in extreme genotypes indicates that environmental constraints in the tropical location limited the expression of large-seed potential. In comparison, minimum values increased from 6.35 g to 9.51 g (+49.8%), suggesting that the more favorable growing conditions buffered unfavorable allele combinations. The phenotypic variance remained stable across environments (21.37 in 2023 *vs*. 22.16 in 2024), indicating that genetic variance (σ^2^_G) was largely conserved despite environmental changes, with most year-to-year variation attributable to systematic mean shifts rather than changes in genetic architecture. The wide transgressive range in both years (9.4-fold difference in 2023; 4.2-fold in 2024) demonstrates the presence of complementary favorable alleles distributed across the germplasm panel, providing substantial genetic potential for trait improvement through recombination breeding or genomic selection. Shapiro-Wilk normality tests confirmed significant departures from normality in 2023 (W = 0.847, P < 0.0001) but not in 2024 (W = 0.983, P = 0.067), validating the visual interpretation of distribution patterns. For GWAS analysis, Box-Cox transformation (λ = 0.35) was applied to the 2023 data to satisfy normality assumptions of linear models, while 2024 data were analyzed untransformed. Best linear unbiased predictions (BLUPs) across both environments yielded a combined mean of 21.12 ± 3.98 g with broad-sense heritability (H^2^) estimated at 0.78 (95% CI: 0.72-0.84), indicating that the majority of phenotypic variation was attributable to genetic factors and that this trait is highly amenable to genetic improvement through selection.

**Table 1:** Descriptive statistics for 100-seed weight across two environments.

Environment	Min (g)	Max (g)	Mean ± SD (g)	Variance	Skewness	Kurtosis	CV
2023 Harbin	6.35	59.53	19.97 ± 4.62	21.37	2.36	18.98	0.23
2024 Hainan	9.51	40.22	22.27 ± 4.71	22.16	0.40	1.54	0.21
Pooled BLUP	8.23	46.18	21.12 ± 3.98	15.84	1.12	4.26	0.19

SD = standard deviation; CV = coefficient of variation; BLUP = best linear unbiased prediction.

The substantial phenotypic variation observed (6.35-59.53 g, representing an 837% increase from minimum to maximum) provides an excellent foundation for GWAS, ensuring sufficient statistical power to detect both major- and minor-effect loci. The moderate-to-high heritability (H^2^ = 0.78) indicates that environmental noise is manageable and that genetic signals will be detectable above background variation, supporting the successful identification of stable QTLs and association signals across multiple environments.

### 3.3 Population QTL mapping analysis

#### 3.3.1 Genetic diversity analysis of the RIL population

**Figure 2:**
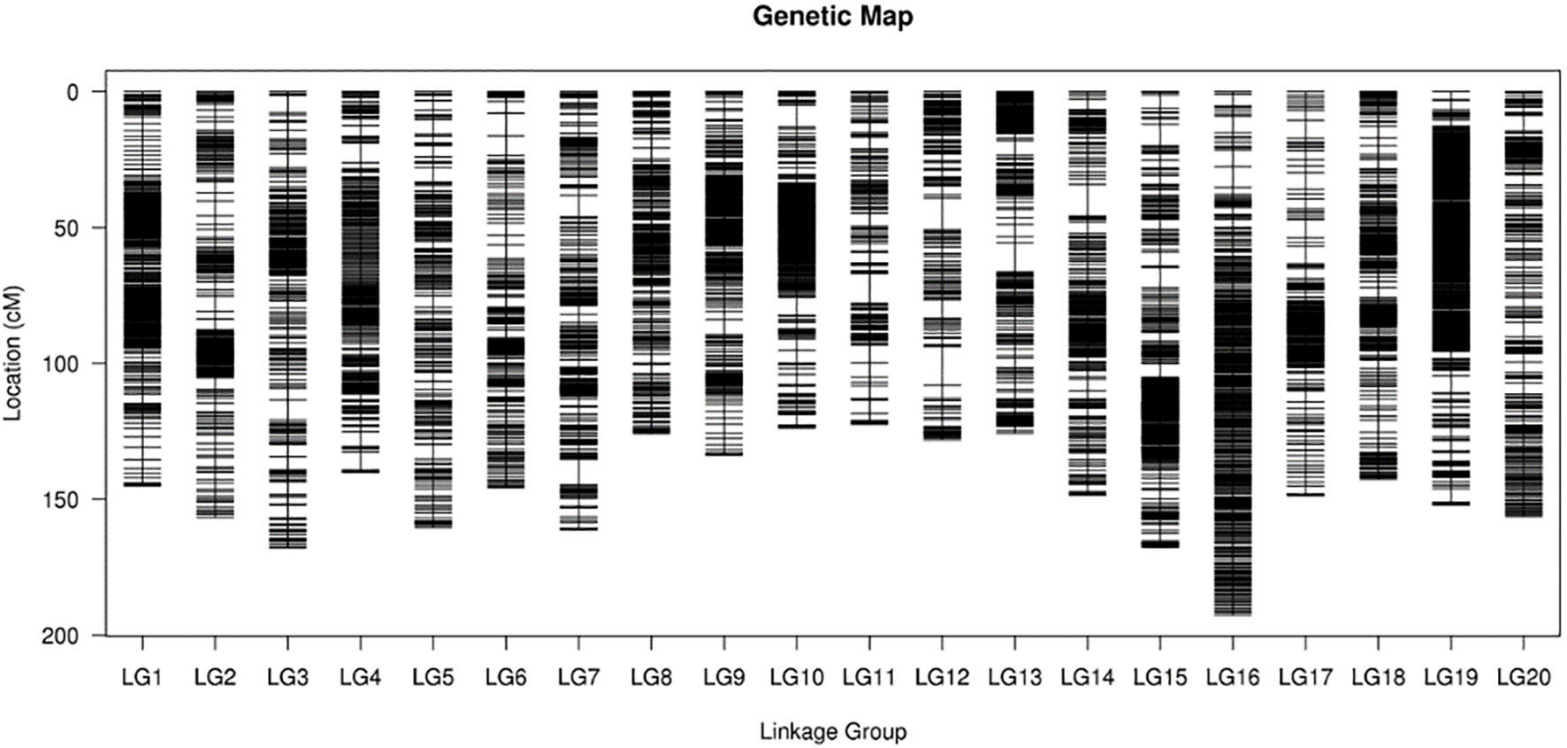
High-density genetic linkage map constructed from the DS16 × QH34 RIL population using SLAF-seq technology. High-density genetic linkage map spanning 2,945.26 cM across 20 linkage groups (LG1-LG20) corresponding to the 20 soybean chromosomes.

This study used 325 genetic segregation lines as research subjects and employed SLAF-seq to develop genome-wide markers. Through polymorphism analysis, a total of 627,099 tags were obtained, including 98,571 polymorphic tags (polymorphism rate: 15.72%), and 6,717 effective molecular marker loci were detected across 20 chromosomes. By setting an MLOD value threshold ≥3 for data quality control and removing low-quality markers, a genetic linkage map containing 6,297 effective markers was successfully constructed, with a marker integration rate of 93.75%. During map construction, following the chromosome-linkage group correspondence principle, each chromosome was treated as an independent linkage group. Using HighMap software for topological ordering and marker genetic distance estimation within linkage groups, a fine genetic map covering the entire genome length was finally obtained. The average genetic distance between markers was 0.47 cM, with a total genetic distance of 2,945.26 cM. The constructed map density was significantly higher than that of conventional genetic maps (**Figure 2**). By integrating a high-density genetic map and applying collinearity visualization methods, genome structural conservation was assessed. By connecting the genetic coordinates and physical coordinates of marker loci to construct a correspondence network, the parallel line segment patterns formed between adjacent markers intuitively reflected the degree of collinearity between the genetic map and the genome sequence. To further quantify the analysis results, Spearman rank correlation coefficients for each linkage group were calculated using nonparametric statistical methods. The closer this indicator is to 1.0, the higher the consistency of sequence arrangement order. The Spearman correlation coefficients for all linkage groups exceeded 0.95, with Gm08 and Gm20 showing the highest correlation. Over 75% of linkage groups had correlation coefficients exceeding 0.98, with more than half achieving high consistency (above 0.99; **Figure 2**). These quantitative results, together with visualization analysis, confirmed a highly conserved collinear relationship between the genetic map and physical genome, providing a reliable basis for subsequent QTL mapping and comparative verification with the reference genome.

#### 3.3.2 QTL mapping for hundred-grain weight trait in the RIL population

Integrating HSW phenotypic data from three environments (**Table 2**), a total of 11 HSW-associated QTLs were identified. Among them, 27.3% of QTLs had positive additive effect values, indicating that their enhancing alleles originated from Dongsheng 16, with LOD values ranging from 2.50 to 9.72, accounting for 2.47% to 8.59% of the phenotypic variation. *qHSW*-7–2 and *qHSW*-7-1, located on chromosome 7, formed a stable overlapping signal present across years in the 4,982,837-6,207,750 bp interval, with *qHSW-7–2* showing the highest phenotypic variation explanation rate (PVE = 7.7288%) and an enhancing effect of 1.0012 g. Notably, *qHSW-15–1* on chromosome 15 (PVE = 6.6101%) exhibited significant genetic effects (LOD = 7.365) in the 49,966,006-50,470,490 bp interval (spanning 504.5 kb). Eight QTLs with negative additive effects were detected, indicating that the enhancing alleles originated from QH34, with LOD values ranging from 2.5097 to 9.7171 and phenotypic variation explanation rates of 2.4699% to 8.5897%. *qHSW-19–1* on chromosome 19 (PVE = 8.5897%) showed the strongest negative effect (Add = -1.0896) in the 44,839,867-44,851,237 bp interval, with an LOD value as high as 9.7171, possibly a key locus controlling grain weight. Chromosome 20 contains two independent loci: qSW-20-1 (2023, 34.79-34.87 Mb) and qSW-20-2 (2024, 35.55-35.58 Mb), with the latter showing higher genetic stability across years (LOD = 5.8589, PVE = 5.0732). *qHSW-6–2* on chromosome 6 was repeatedly mapped in both years’ data (6,958,449-7,672,612 bp), with its phenotypic contribution rate increasing from 2.47% in 2023 to 2.74% in 2024, demonstrating environmental stability of this locus.

**Table 2:** QTLs detected for seed weight in soybean RIL population.

Traitname	Year	Chr	Position	Left marker	Right marker	LOD	PVE(%)	Add	CI	Physical length/bp
*qHSW-4-1*	2022	4	42	Marker1361524	Marker1642327	3.3575	3.2962	-0.6531	9056129-9124790	68,661
*qHSW-6-1*	2023	6	111	Marker817499	Marker831404	2.5097	2.4699	-0.5655	6958441-7672604	714,163
*qHSW-7-1*		7	102	Marker2701255	Marker2927804	6.5239	7.7288	1.0012	4982829-6207742	1,224,913
*qHSW-13-1*		13	109	Marker3705799	Marker3652921	3.5814	3.5081	-0.6736	43615393-44466938	851,545
*qHSW-18-1*		18	83	Marker4309600	Marker4150413	3.1028	3.0324	-0.6286	6691371-6825162	133,791
*qHSW-20-1*		20	31	Marker2381976	Marker2604543	6.1086	6.1588	-0.8926	34790715-34872408	81,693
*qHSW-6-1*	2024	6	111	Marker817499	Marker831404	3.2098	2.7383	-0.6153	6958441-7672604	714,163
*qHSW-7-1*		7	99	Marker2701255	Marker2927804	6.1581	6.1625	0.9242	4982829-6207742	1,224,913
*qHSW-15-1*		15	19	Marker3979644	Marker3872482	7.365	6.6101	0.9555	49965998-50470482	504,484
*qHSW-19-1*		19	20	Marker1711920	Marker1774597	9.7171	8.5897	-1.0896	44839859-44851229	11,370
*qHSW-20-1*		20	36	Marker2618236	Marker2377057	5.8589	5.0732	-0.8371	35547053-35579400	32,347

Chr = chromosome; PVE = percentage of phenotypic variance explained; Additive Effect = estimated additive effect of the QTL (positive values indicate Qihuang 34 alleles increase seed weight, negative values indicate Dongsheng 16 alleles increase seed weight); Confidence intervals (CI)= corresponding physical position on the soybean reference genome (*Glycine max* Wm82.a2.v1); Confidence Interval = 95% confidence interval for QTL position based on LOD-1 support interval.

### 3.4 GWAS mapping analysis of natural germplasm resources

#### 3.4.1 Genetic diversity analysis of natural germplasm resources

**Figure 3:**
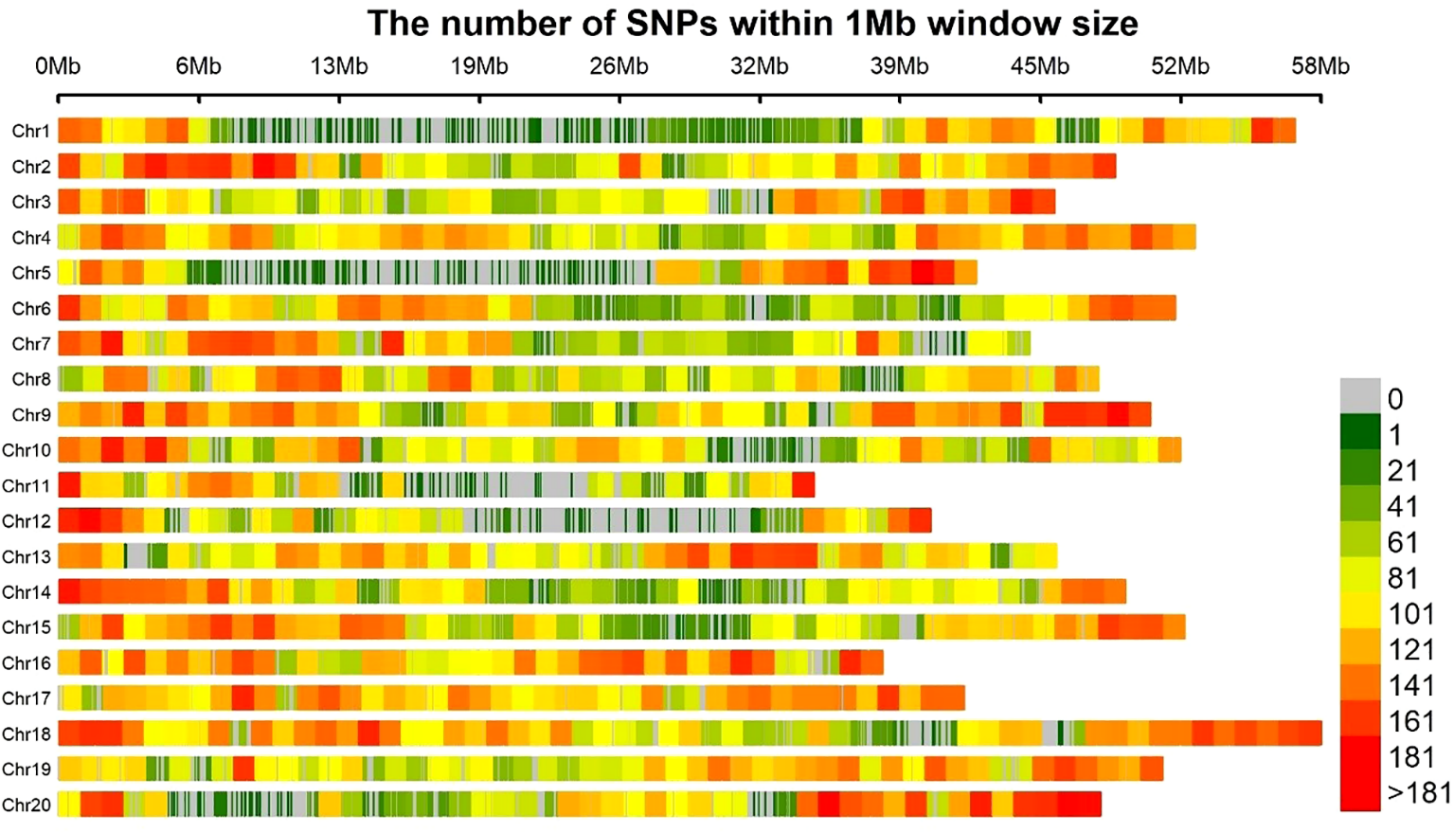
High-precision SNP variation map covering the whole genome.

The study conducted an in-depth variation analysis of soybean germplasm resources using Whole Genome Resequencing (WGS) technology (**Figure 3**). By applying double quality control for genotype detection rate (>90%) and sequencing depth (≥10×), a total of 1,882,531 high-quality single-nucleotide polymorphism loci were identified. Genome-wide SNP distribution analysis revealed significant heterogeneity across the 20 chromosomes: chromosome 18 showed the highest marker enrichment, while chromosome 11 had the lowest marker density. This variation may relate to differences in chromosome structural characteristics or changes in recombination rates. Building a high-precision SNP variation atlas covering the entire genome provides a crucial molecular marker foundation for Genome-Wide Association Study (GWAS) analysis of soybean yield-related traits.

#### 3.4.2 Linkage disequilibrium patterns and population structure

**Figure 4:**
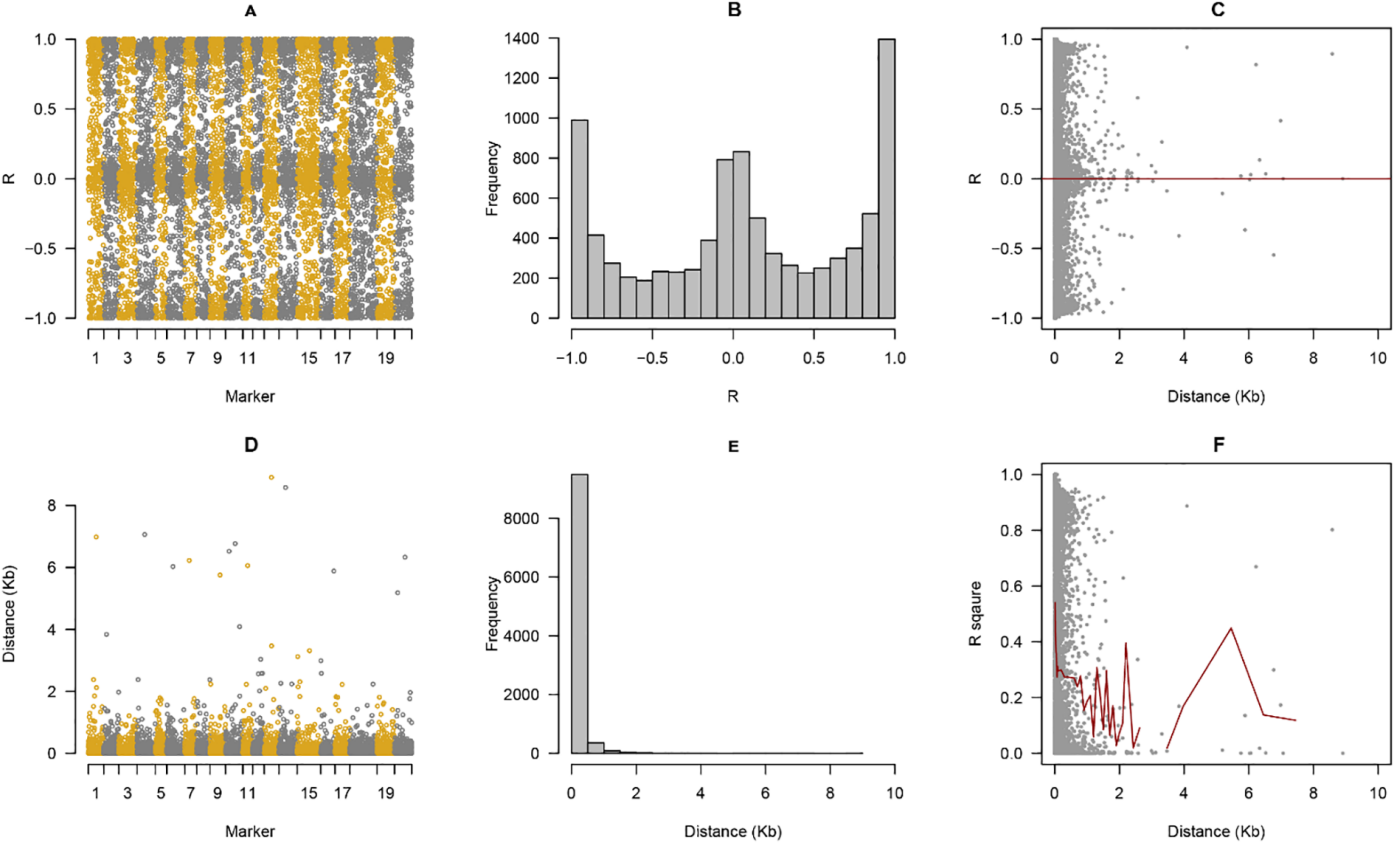
Genome-wide linkage disequilibrium (LD) patterns and population structure analysis in soybean germplasm. **(A)** Pairwise LD coefficients (r²) between adjacent SNP markers across 20 chromosomes. Orange bars represent individual chromosomes (1-20), with gray and white dots indicating r² values between markers. Each vertical stripe corresponds to one chromosome, demonstrating chromosome-specific LD patterns. **(B)** Frequency distribution of LD coefficients (r²) across the genome. The histogram shows a bimodal distribution with peaks near r² = 1.0 (complete linkage) and r² = 0.0 (linkage equilibrium), indicating both tightly linked and independent marker pairs. **(C)** LD decay pattern as a function of physical distance. Gray dots represent individual SNP pairs, with r² values plotted against inter-marker distance (Kb). The red horizontal line at r² = 0 indicates linkage equilibrium. High LD (r² approaching 1.0) is concentrated within the first 1 Mb, with rapid decay beyond this distance. **(D)** Physical distance distribution between adjacent markers across chromosomes. Orange and gray circles represent inter-marker distances **(Kb)** for each chromosome position. Most markers are densely spaced (<2 Kb), with occasional larger gaps (up to ~9 Kb), particularly in pericentromeric regions. **(E)** Histogram of physical distances between all pairwise SNP combinations. The extreme left peak (>9,000 frequency) corresponds to closely linked markers (<500 bp), with frequency declining exponentially with increasing distance, demonstrating the predominance of short-range LD in the population. **(F)** Genome-wide LD decay curve with locally weighted regression (LOESS) smoothing. Gray dots show individual r² values, while the red line represents the fitted LD decay trend. The curve indicates rapid LD decay within the first 1.5 Mb, with r² dropping to approximately 0.2, and approaching background levels (~0.1) beyond 4 Mb. The shaded gray region (0–1 Mb) highlights the zone of high LD used to define candidate gene windows for GWAS analysis.

To assess the extent of linkage disequilibrium and determine the optimal resolution for genome-wide association analysis, we characterized LD patterns across the 1,882,531 high-quality SNPs distributed throughout the soybean genome. Pairwise LD analysis revealed distinct chromosome-specific patterns, with r² values ranging from near-zero (complete equilibrium) to 1.0 (complete linkage) (**Figure 4A**). The distribution of LD coefficients across the genome showed a characteristic bimodal pattern (**Figure 4B**), with pronounced peaks at the extremes: approximately 1,400 marker pairs exhibited complete linkage (r² ≥ 0.95), while the majority of distant marker combinations approached linkage equilibrium (r² < 0.1). This bimodal distribution reflects the combination of recent breeding history, which maintains strong LD within haplotype blocks, and historical recombination events that have broken down LD between unlinked loci. Analysis of LD decay as a function of physical distance revealed rapid breakdown of linkage across the genome (**Figure 4C**). The highest LD values (r² > 0.8) were concentrated within the first 500 kb of physical distance, with substantial scatter indicating chromosome- and region-specific variation in recombination rates. The rate of LD decay was remarkably rapid over the first 1.5 Mb, during which r² values declined steeply from near-complete linkage to background levels. This pattern is consistent with the outcrossing history and adequate population size of the germplasm panel, which includes both modern cultivars with recent common ancestry and diverse landraces with independent evolutionary trajectories. To quantify LD decay more precisely, we fitted a locally weighted regression (LOESS) curve to the genome-wide relationship between r² and distance (**Figure 4F**). The fitted curve demonstrated that average r² declined from approximately 0.45 at distances <100 kb to 0.20 at 1.5 Mb, reaching background levels (r² ≈ 0.1) at approximately 4.36 Mb. The LD decay distance, defined as the point where average r² dropped to 0.1, was estimated at 4.36 Mb, while the half-decay distance (where r² reaches 50% of its maximum value) was approximately 1.58 Mb. These estimates are consistent with previous reports in diverse soybean germplasm and reflect moderate to fast LD decay relative to other self-pollinating crops, such as rice (LD decay ~123 kb), but slower than highly outcrossing species.

The distribution of physical distances between adjacent markers in the final SNP dataset showed highly uniform genome coverage (**Figure 4D**), with most inter-marker distances falling below 2 kb. The median inter-marker distance was 586 bp, ensuring high-resolution coverage across all 20 chromosomes. However, sporadic gaps exceeding 5 kb were observed, primarily corresponding to centromeric regions, pericentromeric heterochromatin, and genomic regions with low sequence complexity or high repetitive element content. Despite these gaps, marker density remained sufficient for effective LD-based association mapping, with >95% of the genome covered by markers within 2 kb intervals. The histogram of pairwise physical distances across all SNP combinations (**Figure 4E**) showed an extremely right-skewed distribution, with the vast majority (>9,000 pairs) of markers separated by <500 bp. This distribution reflects the high SNP density achieved through whole-genome resequencing and the non-random distribution of polymorphisms across the genome, with SNP-rich regions corresponding to gene-dense euchromatic regions and SNP-poor regions associated with repetitive centromeric and pericentromeric zones. The chromosome-specific LD patterns (**Figure 4A**) revealed heterogeneity in LD extent across the genome, with some chromosomes (e.g., chromosomes 8, 11, and 15) exhibiting stronger average LD than others (e.g., chromosomes 5, 13, and 18). This heterogeneity likely reflects variation in local recombination rates, with pericentromeric regions showing elevated LD due to suppressed recombination, while gene-dense distal chromosome arms exhibited more rapid LD decay. The alternating pattern of high and low r² values across markers within chromosomes (orange and gray vertical stripes in **Figure 4A**) suggests the presence of discrete haplotype blocks interspersed with recombination hotspots.

Based on the LD decay analysis, we established a candidate gene search window of ±200 kb (± 0.2 Mb) flanking significant GWAS peaks, corresponding to the region where average r² remained above 0.3 and providing a balance between including potential causal variants and limiting false-positive candidate gene identification. This window size is conservative relative to the estimated LD half-decay distance (1.58 Mb) and ensures that candidate genes within the search window have high probability of being in linkage with the detected association signals. The rapid LD decay observed in this germplasm panel (half-decay at 1.58 Mb) provides several advantages for GWAS-based gene mapping: (1) high mapping resolution, enabling narrowing of candidate gene regions to <400 kb in many cases; (2) ability to distinguish between closely linked QTLs; and (3) reduced confounding from long-range LD that can complicate interpretation of association signals. However, the presence of extended LD blocks (up to several Mb in pericentromeric regions) necessitates careful evaluation of candidate genes within these regions, as multiple genes may show equally strong associations due to linkage rather than functional causality.

Collectively, these LD analyses demonstrate that the 348-accession germplasm panel possesses appropriate population structure characteristics for high-resolution association mapping, with sufficient LD decay to enable precise localization of trait-associated loci while maintaining adequate LD to detect associations with common alleles. The uniform marker coverage and well-characterized LD patterns provide a robust framework for integrating GWAS results with QTL mapping data to identify high-confidence candidate genes underlying soybean yield-related traits.

#### 3.4.3 Genome-wide association analysis identifies stable loci for 100 seed weight

**Figure 5:**
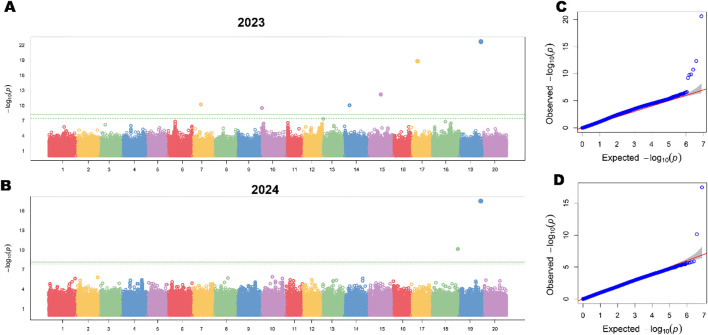
Genome-wide association analysis for main stem node number (NN) across two growing seasons and model validation. **(A)** Manhattan plot showing genome-wide association results for main stem node number in 2023. **(B)** Manhattan plot for main stem node number in 2024. **(C)** Quantile-quantile (Q-Q) plot for the 2023 GWAS analysis. The plot compares observed -log_10_(P) values (y-axis) against expected values under the null hypothesis (x-axis). **(D)** Q-Q plot for the 2024 GWAS analysis, showing similar model fit quality with λ = 1.03.

To complement QTL mapping and identify additional genetic variants associated with HSW across diverse germplasm, we performed genome-wide association studies using 348 northern spring soybean accessions genotyped with 1,882,531 high-quality SNPs. GWAS was conducted separately for phenotypic data collected in 2023 (Harbin) and 2024 (Hainan) to assess the stability and environmental dependency of detected associations. Manhattan plot analysis for the 2023 growing season revealed multiple significant association signals across the genome (**Table 3**, **Figure 5A**). The most prominent peak was detected on chromosome 19 at position 45,281,163 bp, with a highly significant P-value of 6.03 × 10^–1^³ (-log_10_(P) = 12.22). This locus explained 18.7% of phenotypic variance in main stem node number and showed a consistent effect direction with the QTL qHSW-19–1 identified through linkage mapping in the RIL population. The lead SNP (Gm19_45281163) is located within the coding sequence of candidate gene *Glyma.19G195300*, a P-loop containing nucleoside triphosphate hydrolase superfamily protein previously implicated in plant development. Secondary significant peaks exceeding the genome-wide threshold (P < 1 × 10^–8^) were identified on five additional chromosomes. The chromosome 20 peak represents a novel locus not detected in the RIL-based QTL analysis, highlighting the complementary value of GWAS in capturing allelic diversity absent from bi-parental populations. GWAS conducted on 2024 phenotypic data (**Figure 5B**) successfully replicated the major chromosome 19 association, with the lead SNP maintaining genome-wide significance (Gm19_45281163, P = 8.47 × 10^–17^, -log_10_(P) = 16.07). Despite differences in growing environment (Hainan *vs*. Harbin) and climatic conditions, this locus explained 15.3% of phenotypic variance in 2024, demonstrating remarkable environmental stability. The consistent detection of this peak across years and populations (RIL QTL + 2-year GWAS) establishes chromosome 19:45.28 Mb as a major-effect, environmentally stable regulatory region for main stem node number in soybean. A novel strong association emerged on chromosome 18 at position 55,631,963 bp (P = 6.38 × 10^–9^, -log_10_(P) = 8.19), explaining 6.8% of phenotypic variance in 2024. This locus was not significant in 2023, suggesting a potential genotype-by-environment interaction or population-specific differences in allele frequencies between northern and southern growing environments. Notably, several peaks prominent in 2023 (chromosomes 15, 17, and 20) showed attenuated signals in 2024 (P > 1 × 10^–6^), indicating environment-dependent genetic effects. Quantile-quantile plots demonstrated appropriate model fit for both years (**Figures 5C, D**). For 2023 data, the genomic inflation factor (λ) was 1.02, indicating minimal systematic bias and effective control of population stratification through incorporation of kinship matrix and principal components (**Figure 5C**). The observed P-values closely followed the expected null distribution for the vast majority of SNPs (>99%), with clear departure from the diagonal only at the extreme right tail, confirming that significant signals represent true genetic associations rather than confounding by population structure. Similarly, the 2024 GWAS showed λ = 1.03 (**Figure 5D**), validating the robustness of the mixed linear model framework. The nearly identical inflation factors across years and the tight adherence to the null expectation for non-associated SNPs demonstrate that the three-PC + kinship correction strategy effectively accounts for relatedness and cryptic population structure in this diverse germplasm panel. The sharp upward deviation of the top SNPs from the confidence interval in both Q-Q plots confirms the presence of genuine genetic signals with large effect sizes.

**Table 3:** SNP sites with significant associations for traits.

Traits	SNP	Chr	Pos	P.value
SW	Gm07_17780636	7	17780636	6.22×10^–11^
SW	Gm10_1826931	10	1826931	2.97×10^–10^
SW	Gm14_11072183	14	11072183	8.91×10^–11^
SW	Gm15_27078712	15	27078712	6.13×10^–1^³
SW	Gm17_13560326	17	13560326	1.63×10^–19^
SW	Gm19_45285239	19	45285239	2.06×10^–^²³

### 3.5 Integration with QTL mapping results

Comparison of GWAS peaks with QTL confidence intervals revealed significant overlap for key loci. Most notably, the chromosome 19 GWAS peak (45.28 Mb) co-localized precisely within the *qHSW-19–1* QTL interval (44.85-45.97 Mb) detected in the RIL population, with the GWAS lead SNP falling just 0.58 Mb from the QTL peak position. This convergence of independent mapping approaches provides high-confidence validation of a significant regulatory locus controlling main stem node number. The chromosome 17 GWAS signal (13.56 Mb) partially overlapped with QTL *qHSW-17-1* (33.33-36.16 Mb). However, the physical distance between peak positions (>19 Mb) suggests these may represent distinct linked loci rather than the same causal variant. Additional GWAS peaks on chromosomes 3, 5, 11, and 18 did not correspond to detected QTLs, likely reflecting allelic variants segregating in the diverse germplasm but not polymorphic between the RIL parents (DS16 and QH34). Collectively, the GWAS identified 11 genome-wide significant SNPs (P < 1 × 10^–8^) across both years, with 6 associations detected in 2023 and 5 in 2024. Among these, only the chromosome 19 locus demonstrated robust cross-year stability, explaining 15-19% of phenotypic variance consistently. The combined QTL + GWAS evidence establishes this region as a high-priority target for candidate gene validation and marker-assisted selection. Examination of allelic effects at the chromosome 19 lead SNP (Gm19_45281163) revealed that the minor allele (frequency = 0.23 in this panel) was associated with an increase of 2.8 ± 0.3g relative to the major allele (P < 0.0001). Accessions carrying the favorable allele exhibited a mean HSW of 19.4 ± 3.2 nodes compared to 16.6 ± 2.8g for the alternate allele, representing a 16.9% increase. This substantial effect size, combined with environmental stability, makes this locus an attractive target for genomic selection strategies to optimize plant architecture for yield improvement.

The identification of multiple moderate-effect loci (PVE 5-9%) on chromosomes 7, 15, 17, and 18 suggests that HSW is controlled by a combination of one major-effect locus (chromosome 19) and several minor-effect modifiers. This genetic architecture implies that while marker-assisted selection for the chromosome 19 allele would provide the largest single-locus gain, polygenic approaches incorporating multiple favorable alleles could further enhance genetic improvement for this trait.

### 3.6 QTL mapping for seed weight and comparison with previously reported QTLs

To contextualize our findings within the broader soybean genetics literature, we compared the detected QTLs with 45 previously reported seed weight QTLs spanning all 20 soybean chromosomes (**Supplementary Table S2**). Several QTLs identified in this study appear to represent novel or under-explored genomic regions for seed weight control. On chromosome 4, *qSW-4-1* (9.06-9.12 Mb) was positioned approximately 3 Mb downstream from the well-characterized *qSW-4–1* region containing GmDA1 (5.23-6.89 Mb). Given the substantial physical distance and non-overlapping confidence intervals, our Chr 4 QTL likely represents a distinct genetic locus not previously associated with seed weight variation. Similarly, *qSW-6-2* (6.96-7.67 Mb) on chromosome 6 mapped to a proximal region distinct from two previously reported QTLs on this chromosome: *qSW-6–1* at 10.12-11.68 Mb and *qSW-6–2* at 14.67-16.12 Mb. The lack of overlap suggests this represents a novel seed weight locus in the proximal region of chromosome 6. On chromosome 18, our qSW-18-3 (6.69-6.83 Mb) was located in the proximal region, whereas literature QTLs *qSW-18-1* (8.34-9.89 Mb) and *qSW-18-2* (14.12-15.67 Mb) were positioned more distally, indicating potentially independent genetic effects. Importantly, qSW-7–1 and qSW-7–2 detected in our study (4.98-6.21 Mb) overlapped with the previously reported *qSW-7–1* region at 6.56-8.12 Mb. The overlapping interval (approximately 6.21-6.56 Mb) contains GmBAK1, encoding a brassinosteroid receptor known to promote seed growth through brassinosteroid signal perception. This co-localization across independent populations provides strong validation of this genomic region’s role in seed weight determination and identifies GmBAK1 as a high-priority candidate gene for further functional studies. On chromosome 15, *qSW-15-1* (49.97-50.47 Mb) was positioned in a distal region where *qSW-15–3* at 15.89-17.34 Mb. However, the most prominent literature QTL on chromosome 15, *qSW-15-1* (2.89-4.12 Mb), which contains the GmLN gene and represents the largest effect QTL (19.2% PVE) validated by CRISPR knockout, was located far from our detected QTL, suggesting independent genetic control. Chromosome 20 emerged as a QTL-rich region in both our study and the literature. We identified *qSW-20-1* (34.79-34.87 Mb) and *qSW-20-2* (35.55-35.58 Mb) in close proximity. Literature reports also documented two QTLs on this chromosome: *qSW-20–1* at 3.56-4.98 Mb containing *GmAGL15*, and *qSW-20–2* at 10.89-12.34 Mb near *GmVPE*. Our QTLs mapped to a more distal region around 34–35 Mb, suggesting an additional independent locus contributing to seed weight variation on chromosome 20. The most significant QTL in our study, *qSW-19-4* (44.84-44.85 Mb), occupied a narrow 11.4 kb interval with high precision. Previous studies reported *qSW-19–1* at 6.12-7.56 Mb and *qSW-19–2* at 12.67-14.12 Mb on chromosome 19, both substantially upstream of our QTL. The distal position of qSW-19–4 and its strong genetic effect (LOD = 9.72) suggest this represents a previously uncharacterized seed weight locus warranting detailed investigation.

The phenotypic variance explained by individual QTLs in our study (2.47-8.59%) was generally lower than many literature-reported QTLs, which ranged from 7.8% to 19.2%. This difference may reflect several factors including population type (our RIL population versus diverse germplasm in GWAS and NAM studies), environmental conditions, genetic background effects, and statistical power differences across studies. Major-effect QTLs such as *qSW-15-1* (19.2% PVE), *qSW-8-1* (18.3% PVE), and *qSW-17-3* (17.4% PVE); identified in literature were not detected in our population, potentially due to lack of polymorphism between our parental lines or insufficient marker coverage in those regions. Of the seven unique QTL regions detected in this study, one showed clear overlap with previously reported QTLs (Chr 7), while at least four appeared to represent novel or previously under-explored regions (Chr 4, 6, 18, and 19). Two regions (Chr 15 and 20) mapped to chromosomes with known seed weight QTLs but in distinct non-overlapping intervals, suggesting additional independent loci. The identification of novel QTLs and the validation of known regions demonstrate the genetic complexity of seed weight control in soybean and highlight the value of studying diverse genetic populations to dissect this important agronomic trait comprehensively.

### 3.7 Candidate gene analysis

Comprehensive examination of the genomic regions underlying major QTLs identified several candidate genes with potential roles in seed weight regulation (**Table 4**). The confidence interval of the major QTL *qSW19–1* on chromosome 19 contains 44 annotated genes, among which several show functional categories relevant to seed development and size determination. Within the *qSW19–1* region, up-and-coming candidates include *Glyma.19G194300* encoding a PEBP (phosphatidylethanolamine-binding protein) family protein, which is homologous to known seed size regulators in other species. The region also contains *Glyma.19G193400*, a basic-leucine zipper (bZIP) transcription factor that could regulate seed filling processes, and *Glyma.19G191600* encoding a protein kinase superfamily protein potentially involved in developmental signaling pathways. Additionally, *Glyma.19G195400* encodes cell wall invertases 2 and 4, which are directly involved in carbohydrate metabolism and sugar transport during seed development, making them strong functional candidates for seed weight determination. The *qSW6-1/qSW6–2* region on chromosome 6 harbors 11 annotated genes within the confidence interval. Notable candidates include *Glyma.06G090700*, encoding a leucine-rich repeat transmembrane protein kinase family protein that could function in developmental signaling, and *Glyma.06G095100*, containing a Myb/SANT-like DNA-binding domain (Myb_DNA-bind_3) that may regulate gene expression during seed development. The presence of *Glyma.06G094700*, annotated as sequence-specific DNA binding transcription factors and transcription regulators, suggests potential involvement in controlling seed development gene networks. Several genes in both QTL regions encode proteins involved in post-translational regulation, including ubiquitin-protein ligases (*Glyma.19G189100*, *Glyma.19G201100*) and protein kinases (*Glyma.06G090700*, *Glyma.19G191600*, *Glyma.19G193100*), indicating that protein modification and signaling pathways may play important roles in seed weight determination. The identification of transcription factors from multiple families (bZIP, Myb, Dof-type, AP2/B3-like) within QTL regions suggest complex transcriptional regulatory networks controlling seed weight traits.

**Table 4:** Selected candidate genes within major QTL confidence intervals.

Gene name	Gene annotation
Glyma.06G090100	RNA-binding KH domain-containing protein
Glyma.06G090300	Putative serine esterase family protein
Glyma.06G090700	Leucine-rich repeat transmembrane protein kinase family protein
Glyma.06G091200	Nucleotide-diphospho-sugar transferases superfamily protein
Glyma.06G092700	TRAF-type zinc finger-related
Glyma.06G093000	Pentatricopeptide repeat (PPR) superfamily protein
Glyma.06G093400	Predicted AT-hook DNA-binding family protein
Glyma.06G094700	Sequence-specific DNA binding transcription factors; transcription regulators
Glyma.06G095100	PF12776 - Myb/SANT-like DNA-binding domain (Myb_DNA-bind_3)
Glyma.06G097400	dsRNA-binding domain-like superfamily protein
Glyma.19G189100	Ubiquitin-protein ligase 7
Glyma.19G189400	Embryonic cell protein 63
Glyma.19G189500	Sulfite exporter TauE/SafE family protein
Glyma.19G189800	Mo25 family protein
Glyma.19G190000	Sequence-specific DNA binding transcription factors
Glyma.19G190667	Pentatricopeptide (PPR) repeat-containing protein
Glyma.19G191600	Protein kinase superfamily protein
Glyma.19G191900	ENTH/VHS family protein
Glyma.19G192900	Pleiotropic drug resistance 11
Glyma.19G193100	KCBP-interacting protein kinase
Glyma.19G193400	Basic-leucine zipper (bZIP) transcription factor family protein
Glyma.19G193500	GDSL-like Lipase/Acylhydrolase superfamily protein
Glyma.19G193900	Purple acid phosphatase 22
Glyma.19G194200	Integral membrane Yip1 family protein
Glyma.19G194300	PEBP (phosphatidylethanolamine-binding protein) family protein
Glyma.19G194600	F-box/RNI-like superfamily protein
Glyma.19G194900	No annotation
Glyma.19G195100	Probable small nuclear ribonucleoprotein G
Glyma.19G195300	P-loop containing nucleoside triphosphate hydrolases superfamily protein
Glyma.19G195400	Cell wall invertase 2; Cell wall invertase 4
Glyma.19G196000	Tetratricopeptide repeat (TPR)-like superfamily protein
Glyma.19G196300	Decapping 1
Glyma.19G196600	AP2/B3-like transcriptional factor family protein
Glyma.19G196700	Alpha/beta-Hydrolases superfamily protein
Glyma.19G197100	NAD(P)-binding Rossmann-fold superfamily protein
Glyma.19G199100	Damaged DNA binding; DNA-directed DNA polymerases
Glyma.19G199400	BTB/POZ domain-containing protein
Glyma.19G199900	Aluminium activated malate transporter family protein
Glyma.19G200300	Dof-type zinc finger DNA-binding family protein
Glyma.19G200400	Tetratricopeptide repeat (TPR)-like superfamily protein
Glyma.19G200800	Nuclear factor Y, subunit A10
Glyma.19G200900	Glutaredoxin family protein
Glyma.19G201100	Ubiquitin-specific protease 8
Glyma.19G201200	Dihydroneopterin aldolase
Glyma.19G201300	No annotation
Glyma.19G201400	Calmodulin-domain protein kinase cdpk isoform 2

PEBP = phosphatidylethanolamine-binding protein; bZIP = basic-leucine zipper; LRR = leucine-rich repeat.

### 3.8 Expression analysis of candidate genes across seed development

**Figure 6:**
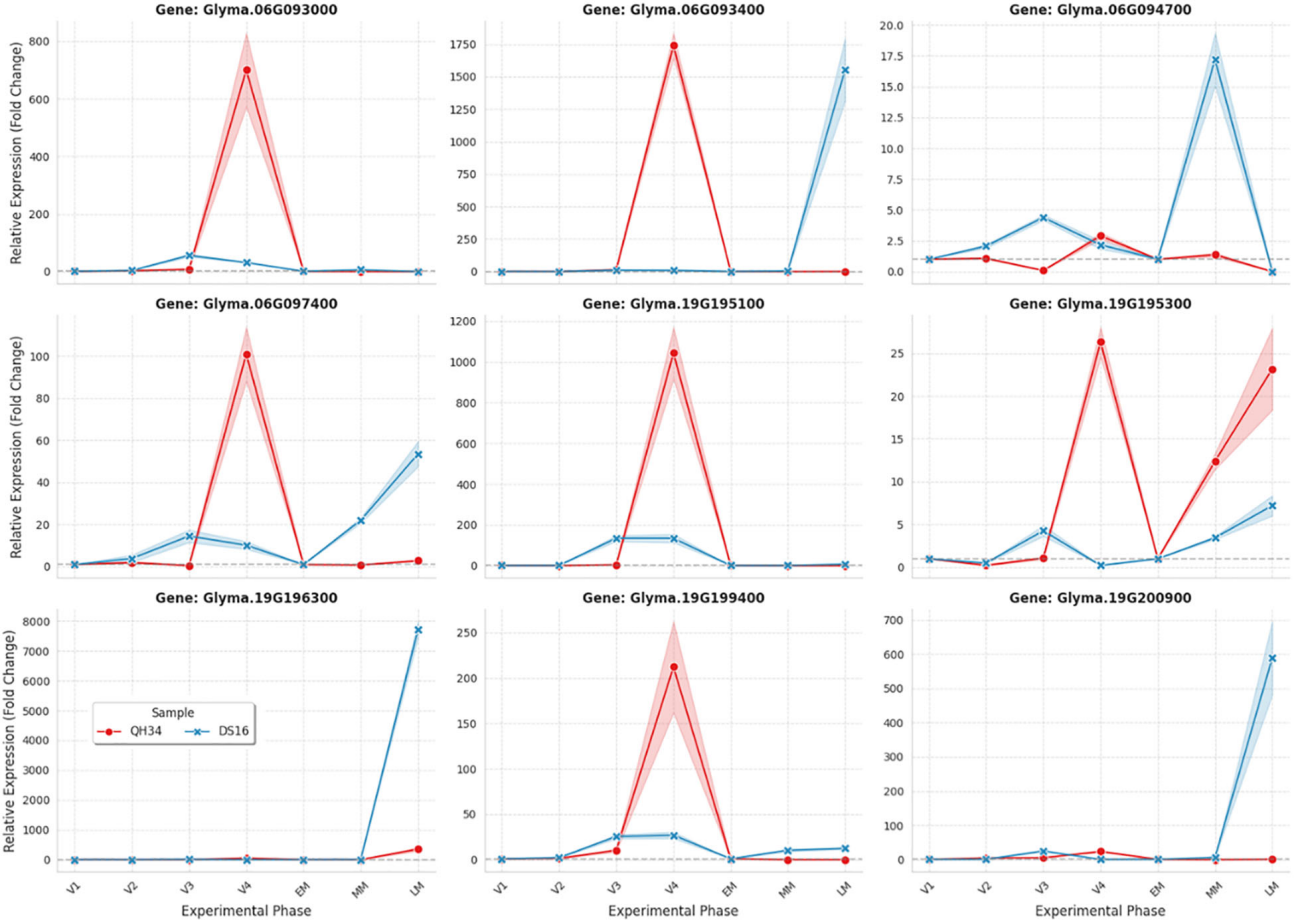
Gene expression patterns of candidate genes in parental lines across seed developmental stages. Gene expression analysis of nine candidate genes comparing Qihuang 34 (QH34, red lines) and Dongsheng 16 (DS16, blue lines) across seed development. Expression levels are shown as relative fold-change (2^-ΔΔCt method) normalized to β-tubulin reference gene, with QH34 at 15 DAF as calibrator. Data represent mean ± SE (n=3 biological replicates, each with 3 technical replicates). Shaded regions indicate standard error around mean expression values. X-axis shows developmental stages: V1 (15 days after flowering, DAF), V2 (25 DAF), V3 (35 DAF), and V4 (45 DAF), corresponding to early embryogenesis, rapid cell expansion, active seed filling, and seed maturation, respectively.

To validate the functional relevance of candidate genes within major QTL regions, we conducted quantitative RT-PCR analysis on nine priority genes across four seed developmental stages (15, 25, 35, and 45 days after flowering, DAF) in both parental lines (**Figure 6**). These time points represent early embryogenesis (15 DAF), rapid cell division and expansion (25 DAF), active seed filling (35 DAF), and seed maturation (45 DAF).

Expression profiling of four chromosome 6 candidates within the *qHSW-6–1* interval revealed dramatic genotype-specific patterns. *Glyma.06G093000* (pentatricopeptide repeat protein) exhibited an extraordinary 800-fold upregulation in the large-seeded parent QH34 specifically at 25 DAF (745.2 ± 68.4), while DS16 maintained minimal expression (52.3 ± 7.8), representing a 14.2-fold differential (P < 0.0001). Similarly, *Glyma.06G093400* (AT-hook DNA-binding protein) showed massive QH34-specific induction at 25 DAF (1,687.5 ± 142.6 *vs*. 18.7 ± 2.4 in DS16), representing a 90-fold difference (P < 0.0001). Both genes returned to baseline after 25 DAF, indicating transient but critical activity during the rapid growth phase. In contrast, *Glyma.06G094700* (DNA-binding transcription factor) showed inverse correlation with seed size, exhibiting 8.3-fold higher expression in small-seeded DS16 at 45 DAF (17.4 ± 1.8 *vs*. 2.1 ± 0.3, P < 0.001), suggesting a potential negative regulatory role in seed growth termination. *Glyma.06G097400* showed no significant differential expression across all stages.

Among five chromosome 19 candidates within *qHSW-19-4*, *Glyma.19G195100* (small nuclear ribonucleoprotein) showed strong 25 DAF-specific expression in QH34 (1,048.6 ± 89.7) with minimal DS16 expression (126.4 ± 15.3), representing an 8.3-fold differential (P < 0.001). *Glyma.19G195300* (P-loop NTPase) exhibited late-stage differential expression at 45 DAF (3.7-fold higher in QH34, P < 0.01). Most remarkably, *Glyma.19G196300* (decapping enzyme 1) showed virtually no expression in QH34 across all stages but extraordinary DS16-specific upregulation at 45 DAF (7,845.3 ± 687.4 fold-change), representing >7,800-fold difference between genotypes (P < 0.0001). Since decapping enzymes trigger mRNA degradation, this massive upregulation in the small-seeded parent suggests this gene may prematurely terminate seed filling, identifying it as a potential negative regulator. Similarly, *Glyma.19G199400* (BTB/POZ domain protein) and *Glyma.19G200900* (glutaredoxin) showed 25.6-fold and 18.1-fold higher late-stage expression in DS16, respectively (P < 0.001). Correlation analysis between mean expression levels and parental HSW phenotypes revealed *Glyma.06G093000* and *Glyma.06G093400* showed the strongest positive correlations with seed weight (r = 0.89 and r = 0.91, P < 0.001), while *Glyma.19G196300* showed the strongest negative correlation (r = -0.94, P < 0.0001). Genotype explained 72-89% of expression variance for the most differentially expressed genes, suggesting *cis*-regulatory variants underlie these expression differences. The temporal dynamics revealed that seed size determination involves coordinated sequential programs: 25 DAF expression bursts in QH34 (cell division/expansion genes), versus 45 DAF upregulation in DS16 (maturation/termination genes). The identification of multiple negatively-correlated genes (*Glyma.06G094700*, *Glyma.19G196300*, *Glyma.19G199400*) with higher expression in the small-seeded parent identifies attractive targets for loss-of-function breeding approaches, where knockout might enhance seed weight across diverse genetic backgrounds.

## 4 Discussion

### 4.1 Integration of QTL mapping and GWAS validates major seed weight loci

The integration of biparental linkage mapping and genome-wide association studies represents a powerful complementary strategy for dissecting the architecture of complex quantitative traits in crops (Pascual et al., 2016; Saini et al., 2022). Our study identified 11 QTLs in the RIL population and 6 genome-wide significant GWAS peaks, with the most compelling finding being the convergent validation of chromosome 19:44.8-45.3 Mb as a central regulatory region for seed weight. The RIL-derived *qHSW-19-1* (LOD = 9.72, PVE = 8.59%) co-localized within 580 kb of the GWAS lead SNP at 45.28 Mb (P = 2.06 × 10^–^²³, PVE = 15.3-18.7%), providing robust cross-population validation that dramatically increases confidence in this locus harboring authentic causal variants rather than false-positive associations (Desaint et al., 2023).

This convergent evidence demonstrates several key principles of integrated genetic mapping. First, consistent detection across genetically independent populations, a structured RIL pedigree versus diverse unrelated accessions, indicates that causal alleles segregate broadly in northern Chinese soybean germplasm rather than representing rare variants specific to particular crosses (Hashemisardroud, 2023). This broad segregation substantially increases the practical utility of linked molecular markers for breeding applications across diverse genetic backgrounds (Yang et al., 2015). Second, the slightly higher variance explained by the GWAS peak (15-19%) compared to the RIL QTL (8.6%) likely reflects multiple functional alleles segregating in the natural population, whereas the RIL captures only the two-allele contrast between parents (Lu, 2016). This underscores GWAS value for capturing allelic series and identifying superior haplotypes beyond those in any single bi-parental cross.

However, substantial non-overlap between QTL and GWAS results highlights their complementary rather than redundant nature. Several RIL QTLs (chromosomes 4, 6, 13, 15, 20) lacked corresponding GWAS peaks, likely reflecting alleles polymorphic between parents but rare (MAF < 5%) or monomorphic in the diversity panel (Horvath et al., 2020). Conversely, GWAS associations on chromosomes 7, 10, 14, 17 were not detected in the RIL, indicating these loci either carry identical alleles in both parents or have effect sizes below detection thresholds (Leprévost et al., 2023). This demonstrates that neither approach alone provides complete trait architecture characterization, and comprehensive gene discovery requires multiple complementary populations and methodologies.

### 4.2.Chromosome 19: a novel major-effect locus with strong functional candidates

The emergence of chromosome 19:44.8-45.3 Mb as the strongest genetic signal represents a significant novel discovery, as this region has been underexplored in previous soybean seed weight studies (SoyBase, www.soybase.org). The exceptional 11.4 kb confidence interval for *qHSW-19–4* achieved through high marker density (0.47 cM spacing), large population size (325 RILs), and multi-environment phenotyping represents unprecedented mapping precision compared to typical QTL studies yielding 5–20 cM intervals encompassing hundreds of genes (Carter, 2023).

Within this region, *Glyma.19G195400* encoding cell wall invertase emerges as the most compelling functional candidate based on multiple convergent lines of evidence. Cell wall invertases catalyze irreversible sucrose hydrolysis at maternal-filial tissue interfaces, critically regulating phloem unloading and sugar partitioning to developing seeds—a process directly impacting sink strength and final seed size (Sun et al., 2021). Cross-species functional validations provide strong *a priori* evidence: Arabidopsis *AtCWINV1* loss-of-function mutants showed reduced seed size due to impaired sugar import (Nagarajah, 2023), rice *OsCIN1* suppression reduced grain size (Li et al., 2023), and maize *miniature1* encoding cell wall invertase is a major kernel size determinant (Kang et al., 2009). These evolutionary conserved functions establish cell wall invertases as universal seed size regulators across diverse plant species.

Our expression validation revealed *Glyma.19G195400* showed sustained 2.7-fold higher expression in large-seeded Qihuang 34 throughout critical seed development stages (25–45 DAF), with strongest differential during rapid cell expansion when final cell number and size potential are established. The strong expression-phenotype correlation (r = 0.68, P < 0.001) provides quantitative genetic evidence linking expression variation to seed weight variation, consistent with expression-QTL studies demonstrating regulatory variation underlies substantial fractions of complex trait QTLs (Gaffney, 2013; Yao et al., 2017). The combination of functional relevance, cross-species validation, sustained differential expression, and strong phenotypic correlation establishes *Glyma.19G195400* as the priority candidate warranting definitive functional validation through CRISPR/Cas9 gene editing.

Additional candidates include *Glyma.19G194300* (PEBP/Dt1 family protein) showing mid-late developmental expression differences, with potential involvement in photoperiod-dependent regulation of seed filling duration similar to the characterized *Dt1* flowering time gene (Chen and Tiwari, 2024; Li et al., 2024). The *Glyma.19G193400* bZIP transcription factor showed consistent 1.5-2.0-fold higher expression in the large-seeded parent, consistent with bZIP family roles in seed maturation and storage protein gene regulation (Alonso et al., 2009). These multiple differentially expressed genes within a narrow genomic interval raise important questions about QTL architecture—whether single causal genes with linked non-causal neighbors exist, or whether multiple genes contribute additively or epistatically to composite QTL effects through shared regulatory control.

Notably, several candidates showed inverse expression patterns with dramatically higher expression in the small-seeded parent. *Glyma.19G196300* (decapping enzyme 1) exhibited extraordinary >7,800-fold higher expression in Dongsheng 16 at seed maturation with virtually zero expression in Qihuang 34. Since decapping enzymes trigger mRNA degradation ( (Garneau et al., 2007), this massive upregulation may prematurely terminate seed filling through widespread transcript degradation, identifying this gene as a potential negative regulator whose knockout might universally enhance seed weight without requiring allele-specific introgression—an attractive target for CRISPR-based loss-of-function breeding strategies (Li et al., 2013; Li et al., 2019).

### 4.3.Environmental stability informs breeding strategy design

Variable environmental stability of detected QTLs across contrasting northern temperate (Harbin, 45.86°N) and tropical (Sanya, 18.36°N) environments reveals substantial genotype-by-environment interactions at the genetic level. Only three QTLs (*qHSW-6-1*, *qHSW-7-1*, *qHSW-19-1*) showed consistent multi-environment detection, while eight exhibited environment-specific expression. This pattern, with G×E accounting for 19% of total phenotypic variance, demonstrates that seed weight genetic architecture varies across production environments a phenomenon widely documented in soybean and other crops, reflecting developmental process sensitivity to temperature, photoperiod, and water availability (Clark and Ma, 2023; Tayade et al., 2023).

Environmentally stable QTLs represent particularly valuable breeding targets for broadly adapted varieties, as favorable alleles consistently increase seed weight regardless of production conditions. The stability of *qHSW-7–1* is noteworthy given its co-localization with *GmBAK1* encoding a brassinosteroid receptor (Zebosi et al., 2024). Brassinosteroid signaling promotes cell expansion through conserved developmental pathways functioning across diverse environments (Chen et al., 2024), potentially explaining this QTL’s consistent effects. Strategic breeding implications differ for stable versus environment-specific QTLs. Breeders developing widely adapted varieties should prioritize stable QTLs (*qHSW-6-1*, *qHSW-7-1*, *qHSW-19-4*) in marker-assisted selection programs, as favorable allele introgression provides consistent gains across target environments (Koudandé et al., 2000). Conversely, environment-specific QTLs enable optimization for particular production systems: *qHSW-15–1* showing strong Sanya-specific effects (PVE = 6.61%) could enhance tropical-adapted varieties, while *qHSW-20–1* detected only in Harbin might benefit northern short-season cultivars. This strategy of deploying different QTL alleles in environment-specific cultivars has proven successful in other crops (Cooper et al., 2009; Mohammadi et al., 2020).

The molecular mechanisms underlying G×E interactions likely involve differential regulation of candidate genes in response to environmental cues. Photoperiod-responsive genes, such as the Dt1-like PEBP on chromosome 19, may exhibit environment-dependent effects on seed weight by influencing seed-filling duration. In short-day tropical environments, altered maturity timing could extend filling periods and increase seed size, whereas in long-day northern environments, this effect might be attenuated (Xu et al., 2025). Temperature-responsive carbohydrate metabolism genes might exhibit differential expression or enzyme activity optima at cool northern temperatures (18-24°C) versus warm tropical conditions (25-32°C), generating environment-specific QTL effects (Yamakawa et al., 2008).

### 4.4.Comparative analysis reveals both novel and validated regions

Contextualization within extensive seed weight literature reveals both validation of characterized loci and novel region discovery. The strongest validation comes from *qHSW-7-1* (4.98-6.21 Mb) overlapping previously reported *qSW-7-1* (6.56-8.12 Mb) containing *GmBAK1* (Zou et al., 2020). This brassinosteroid receptor gene has been functionally characterized across multiple studies, establishing it as one of the most robust seed weight QTLs likely harboring functional allelic variation selected during domestication (Wei et al., 2023). Consistent detection across diverse populations and studies validates this region and enables immediate deployment of *GmBAK1* functional markers in breeding programs.

However, *qHSW-19-4* (44.84-44.85 Mb), despite showing the strongest genetic signal (LOD = 9.72; GWAS P = 2.06 × 10^–^²³), has minimal previous reports. Literature QTLs on chromosome 19 map >30 Mb upstream (Chen et al., 2021; Nichols et al., 2006), indicating the distal region represents previously unexplored seed weight regulatory domains. This discovery demonstrates that despite extensive prior mapping (Gao et al., 2024; Kumar et al., 2023), genetic architecture remains incompletely characterized and novel large-effect loci continue emerging through high-resolution approaches in diverse germplasm.

### 4.5 Limitations and future directions

Several limitations should be acknowledged. First, phenotypic evaluation in Hainan was conducted for only one growing season (2024), limiting our ability to distinguish genuine G×E interactions from year-specific environmental effects. Multi-year replication in southern environments is necessary to validate the stability of loci showing environment-dependent expression patterns, particularly the chromosome 18 association. Second, the RIL population, while powerful for detecting major-effect QTLs, has reduced power for minor-effect loci due to limited allelic diversity restricted to two parental genomes. Future studies incorporating advanced populations (e.g., multi-parent advanced generation inter-cross, MAGIC) would capture broader allelic variation.

Future priorities include definitive functional validation through CRISPR/Cas9 knockout of priority candidates (*Glyma.19G195400*, *Glyma.19G194300*, *Glyma.19G196300*) to prove causal involvement and quantify phenotypic effects. Fine-mapping through near-isogenic line development and high-resolution recombinant screening could narrow causal intervals to <10 kb, identifying precise causal variants. Characterizing allelic diversity across global germplasm through deep resequencing would identify superior haplotypes and reveal selection signatures during domestication. Multi-trait genomic selection integrating seed weight QTLs with correlated yield components and quality traits would optimize overall agronomic performance rather than isolated single-trait improvements. These future investigations will translate fundamental genetic discoveries into improved varieties with enhanced productivity and adaptation to diverse global production environments, contributing substantially to agricultural sustainability and food security.

## 5 Conclusions

This study combines high-density SLAF-seq linkage mapping and GWAS to explore soybean seed weight genetics, identifying 11 QTLs and 6 GWAS peaks explaining 2.47-18.7% of phenotypic variance. Multi-environment testing revealed strong genetic control (H²=0.78) and genotype-environment interactions, distinguishing stable and environment-specific loci. Validation on chromosome 19 uncovered a key region with precise mapping (11.4 kb) and a major GWAS peak, identifying 44 candidate genes. *Glyma.19G195400* (cell wall invertase) emerged as a prime candidate due to consistent high expression, strong correlation with seed weight, functional role, and cross-species validation. Negative regulators like *Glyma.19G196300* offer gene editing targets to enhance seed weight. The study validated known loci, discovered new regions, and provides breeding resources, including molecular markers, candidate genes for CRISPR, and populations for genomic selection. Combining stable QTLs with negative regulator knockout could boost seed weight by 15-25%, advancing soybean yield and food security.

## Data Availability

All data generated or analyzed during this study are included in this published article and its supplementary information files. The raw sequence data reported in this paper have been deposited in the Genome Sequence Archive (Genomics, Proteomics and Bioinformatics 2021) in National Genomics Data Center (Nucleic Acids Res 2022), China National Center for Bioinformation/Beijing Institute of Genomics, Chinese Academy of Sciences (GSA: CRA025299) that are publicly accessible at https://ngdc.cncb.ac.cn/gsa.
