# Supplementary material for: Integrating linkage mapping and GWAS reveals novel genetic architecture of seed weight in soybean (Glycine max L.)
Source: Front Plant Sci. 2026 Jan 5;16:1711905. doi: 10.3389/fpls.2025.1711905 (PMC12812886; doi:10.3389/fpls.2025.1711905)
Supplement: Supplementary file 1 [file Table1.docx]

**Table S1. Primer sequences for qRT-PCR analysis of candidate genes**

| **Gene ID** | **Gene Name** | **Forward Primer (5'-3')** | **Reverse Primer (5'-3')** | **Product Size (bp)** | **Tm (°C)** |
| --- | --- | --- | --- | --- | --- |
| Glyma.19G194300 | PEBP Family protein | TGACAAGGCTGAGTTCGTCA | CATGGTGAAGCCGTCAATGT | 145 | 60.2 |
| Glyma.19G195400 | Cell wall invertase | AGGCTGTGAACTTGGTGGAT | TCCTGTAGCCACTGGATTCC | 162 | 59.8 |
| Glyma.19G193400 | bZIP transcription factor | CAAGAGGCAGCTGAACTCGT | GTCCACATAGCGCTGTAGCA | 138 | 60.5 |
| Glyma.19G191600 | Protein kinase superfamily | TCCGAAGTGGTCAAGCTGAA | AGTGCTTCACGGTGAAGTTG | 154 | 59.9 |
| Glyma.06G095100 | Myb DNA-binding domain | GCTCAAGAGCAGCAACCAAT | TTGGTGACGTGAGCATAGGA | 149 | 60.1 |
| Glyma.06G090700 | LRR transmembrane protein kinase | AGGAGTACCCGACGAACAAG | CGTCTCCTCCACCAAGATCA | 171 | 59.7 |
| Glyma.06G094700 | DNA-binding transcription factors | GAACCTGGTGAAGCTGTTGG | CTCCTGGTTGACCTGCAAAT | 142 | 60.3 |
| Glyma.05G157200 | β-tubulin (reference gene) | TGAGCAGTTCACGGCTATGA | CGAACATCTCCTGACCCTGT | 158 | 60.0 |

*Tm = melting temperature; LRR = leucine-rich repeat; PEBP = phosphatidylethanolamine-binding protein; bZIP = basic-leucine zipper*
