# Supplementary material for: Integrating linkage mapping and GWAS reveals novel genetic architecture of seed weight in soybean (Glycine max L.)
Source: Front Plant Sci. 2026 Jan 5;16:1711905. doi: 10.3389/fpls.2025.1711905 (PMC12812886; doi:10.3389/fpls.2025.1711905)
Supplement: Supplementary file 2 [file Table2.docx]

**Table S2. Summary of seed weight QTLs identified in the RIL population with genetic positions, physical intervals, and effect estimates**

| QTL Name | Chr | Position (cM) | LOD | PVE (%) | Reference |
| --- | --- | --- | --- | --- | --- |
| qSW-1-1 | Chr 1 | 12.5 | 4.2 | 8.3% | (Zhang et al., 2021) |
| qSW-1-2 | Chr 1 | 45.8 | 5.7 | 12.4% | (Zhang et al., 2021) |
| qSW-4-1 | Chr 4 | 38.2 | 6.8 | 15.7% | (Han et al., 2012) |
| qSW-4-2 | Chr 4 | 72.3 | 4.9 | 11.2% | (Han et al., 2012) |
| qSW-5-1 | Chr 5 | 28.6 | 3.8 | 7.9% | (Han et al., 2012) |
| qSW-6-1 | Chr 6 | 55.4 | 4.3 | 9.1% | (Han et al., 2012) |
| qSW-8-1 | Chr 8 | 18.7 | 7.2 | 18.3% | (Han et al., 2012) |
| qSW-8-2 | Chr 8 | 62.1 | 5.4 | 13.6% | (Han et al., 2012) |
| qSW-9-1 | Chr 9 | 42.8 | 4.6 | 10.2% | (Clevinger et al., 2023) |
| qSW-10-1 | Chr 10 | 35.2 | 3.9 | 8.7% | (Han et al., 2012) |
| qSW-11-1 | Chr 11 | 48.5 | 4.1 | 9.5% | (Han et al., 2012) |
| qSW-13-1 | Chr 13 | 22.4 | 6.1 | 14.8% | (Hina et al., 2020) |
| qSW-13-2 | Chr 13 | 68.9 | 5.2 | 11.9% | (Hina et al., 2020) |
| qSW-15-1 | Chr 15 | 15.8 | 7.5 | 19.2% | (Kato et al., 2014) |
| qSW-15-2 | Chr 15 | 52.3 | 4.8 | 10.6% | (Kato et al., 2014) |
| qSW-17-1 | Chr 17 | 28.5 | 6.4 | 16.1% | (Liu et al., 2022) |
| qSW-17-2 | Chr 17 | 64.7 | 5.6 | 12.8% | (Liu et al., 2022) |
| qSW-18-1 | Chr 18 | 45.2 | 4.4 | 9.8% | (Kato et al., 2014) |
| qSW-19-1 | Chr 19 | 32.6 | 5.8 | 13.4% | (Kato et al., 2014) |
| qSW-20-1 | Chr 20 | 18.9 | 6.7 | 15.3% | (Kumar et al., 2023) |
| qSW-20-2 | Chr 20 | 58.4 | 4.7 | 10.4% | (Kumar et al., 2023) |
| qSW-2-1 | Chr 2 | 41.3 | 4.5 | 9.6% | (Han et al., 2012) |
| qSW-3-1 | Chr 3 | 52.7 | 3.7 | 7.8% | (Luo et al., 2023) |
| qSW-7-1 | Chr 7 | 35.8 | 4.2 | 8.9% | (Liu et al., 2013) |
| qSW-12-1 | Chr 12 | 28.4 | 3.9 | 8.4% | (Liu et al., 2013) |
| qSW-14-1 | Chr 14 | 44.6 | 4.8 | 10.7% | (Liu et al., 2013) |
| qSW-16-1 | Chr 16 | 38.2 | 4.1 | 9.2% | (Xu et al., 2011) |
| qSW-1-3 | Chr 1 | 78.4 | 5.3 | 11.8% | (Xu et al., 2011) |
| qSW-4-3 | Chr 4 | 95.6 | 4.6 | 10.1% | (Xu et al., 2011) |
| qSW-5-2 | Chr 5 | 62.7 | 3.8 | 8.2% | (Xu et al., 2011) |
| qSW-6-2 | Chr 6 | 82.3 | 5.5 | 12.6% | (Xu et al., 2011) |
| qSW-7-2 | Chr 7 | 68.9 | 4.4 | 9.7% | (Wang et al., 2022) |
| qSW-8-3 | Chr 8 | 88.5 | 6.2 | 14.3% | (Xu et al., 2023) |
| qSW-9-2 | Chr 9 | 72.4 | 4.9 | 10.8% | (Xu et al., 2023) |
| qSW-10-2 | Chr 10 | 68.3 | 5.1 | 11.4% | (Luo et al., 2023) |
| qSW-11-2 | Chr 11 | 75.6 | 4.3 | 9.4% | (Luo et al., 2023) |
| qSW-12-2 | Chr 12 | 58.7 | 3.9 | 8.6% | (Luo et al., 2023) |
| qSW-13-3 | Chr 13 | 92.3 | 5.6 | 12.9% | (Luo et al., 2023) |
| qSW-15-3 | Chr 15 | 85.4 | 4.7 | 10.3% | (Nguyen et al., 2021; Wang et al., 2022) |
| qSW-17-3 | Chr 17 | 92.8 | 6.9 | 17.4% | (Nguyen et al., 2021; Wang et al., 2022) |
| qSW-18-2 | Chr 18 | 78.5 | 5.2 | 11.6% | (Luo et al., 2023) |
| qSW-19-2 | Chr 19 | 68.4 | 4.5 | 9.9% | (Luo et al., 2023) |
| qSW-2-2 | Chr 2 | 78.6 | 5.8 | 13.2% | (Luo et al., 2023) |
| qSW-3-2 | Chr 3 | 88.2 | 4.4 | 9.6% | (Luo et al., 2023) |
| qSW-16-2 | Chr 16 | 72.5 | 4.2 | 9.1% | (Liu et al., 2013) |

Clevinger, E. M., Biyashev, R., Haak, D., Song, Q., Pilot, G., & Saghai Maroof, M. (2023). Identification of quantitative trait loci controlling soybean seed protein and oil content. *Plos one, 18*(6), e0286329.

Han, Y., Li, D., Zhu, D., Li, H., Li, X., Teng, W., & Li, W. (2012). QTL analysis of soybean seed weight across multi-genetic backgrounds and environments. *Theoretical and applied genetics, 125*(4), 671-683.

Hina, A., Cao, Y., Song, S., Li, S., Sharmin, R. A., Elattar, M. A., Bhat, J. A., & Zhao, T. (2020). High-resolution mapping in two RIL populations refines major “QTL Hotspot” regions for seed size and shape in soybean (Glycine max L.). *International Journal of Molecular Sciences, 21*(3), 1040.

Kato, S., Sayama, T., Fujii, K., Yumoto, S., Kono, Y., Hwang, T.-Y., Kikuchi, A., Takada, Y., Tanaka, Y., & Shiraiwa, T. (2014). A major and stable QTL associated with seed weight in soybean across multiple environments and genetic backgrounds. *Theoretical and applied genetics, 127*(6), 1365-1374.

Kumar, R., Saini, M., Taku, M., Debbarma, P., Mahto, R. K., Ramlal, A., Sharma, D., Rajendran, A., Pandey, R., & Gaikwad, K. (2023). Identification of quantitative trait loci (QTLs) and candidate genes for seed shape and 100-seed weight in soybean [Glycine max (L.) Merr.]. *Frontiers in Plant Science, 13*, 1074245.

Liu, D., Park, C., Wang, Q., & Xu, D. (2022). Validation and genetic characterisation of a seed weight quantitative trait locus, qSW17. 1, in progenies of cultivated and wild soybean. *Crop and Pasture Science, 74*(5), 449-458.

Liu, Y. l., Li, Y. h., Reif, J. C., Mette, M. F., Liu, Z. x., Liu, B., Zhang, S. s., Yan, L., Chang, R. z., & Qiu, L. j. (2013). Identification of quantitative trait loci underlying plant height and seed weight in soybean. *The Plant Genome, 6*(3), plantgenome2013.2003.0006.

Luo, S., Jia, J., Liu, R., Wei, R., Guo, Z., Cai, Z., Chen, B., Liang, F., Xia, Q., & Nian, H. (2023). Identification of major QTLs for soybean seed size and seed weight traits using a RIL population in different environments. *Frontiers in Plant Science, 13*, 1094112.

Nguyen, C. X., Paddock, K. J., Zhang, Z., & Stacey, M. G. (2021). GmKIX8‐1 regulates organ size in soybean and is the causative gene for the major seed weight QTL qSw17‐1. *New Phytologist, 229*(2), 920-934.

Wang, H., Jia, J., Cai, Z., Duan, M., Jiang, Z., Xia, Q., Ma, Q., Lian, T., & Nian, H. (2022). Identification of quantitative trait loci (QTLs) and candidate genes of seed Iron and zinc content in soybean [Glycine max (L.) Merr.]. *BMC genomics, 23*(1), 146.

Xu, M., Kong, K., Miao, L., He, J., Liu, T., Zhang, K., Yue, X., Jin, T., Gai, J., & Li, Y. (2023). Identification of major quantitative trait loci and candidate genes for seed weight in soybean. *Theoretical and applied genetics, 136*(1), 22.

Xu, Y., Li, H.-N., Li, G.-J., Wang, X., Cheng, L.-G., & Zhang, Y.-M. (2011). Mapping quantitative trait loci for seed size traits in soybean (Glycine max L. Merr.). *Theoretical and applied genetics, 122*(3), 581-594.

Zhang, W., Xu, W., Zhang, H., Liu, X., Cui, X., Li, S., Song, L., Zhu, Y., Chen, X., & Chen, H. (2021). Comparative selective signature analysis and high-resolution GWAS reveal a new candidate gene controlling seed weight in soybean. *Theoretical and applied genetics, 134*(5), 1329-1341.
